# Non-centrosomal nucleation mediated by augmin organizes microtubules in post-mitotic neurons and controls axonal microtubule polarity

**DOI:** 10.1038/ncomms12187

**Published:** 2016-07-13

**Authors:** Carlos Sánchez-Huertas, Francisco Freixo, Ricardo Viais, Cristina Lacasa, Eduardo Soriano, Jens Lüders

**Affiliations:** 1 Institute for Research in Biomedicine (IRB Barcelona), The Barcelona Institute of Science and Technology, Baldiri Reixac 10, 08028 Barcelona, Spain; 2 Department of Cell Biology, Physiology and Immunology, Faculty of Biology and INUB, University of Barcelona, Barcelona 08028, Spain; 3 Centro de Investigación Biomédica en Red sobre Enfermedades Neurodegenerativas (CIBERNED), ISCIII, Madrid 28031, Spain; 4 Vall d'Hebron Institute of Research, Barcelona 08035, Spain; 5 Institució Catalana de Recerca i Estudis Avançats (ICREA), Barcelona 08010, Spain

## Abstract

Neurons display a highly polarized microtubule network that mediates trafficking throughout the extensive cytoplasm and is crucial for neuronal differentiation and function. In newborn migrating neurons, the microtubule network is organized by the centrosome. During neuron maturation, however, the centrosome gradually loses this activity, and how microtubules are organized in more mature neurons remains poorly understood. Here, we demonstrate that microtubule organization in post-mitotic neurons strongly depends on non-centrosomal nucleation mediated by augmin and by the nucleator γTuRC. Disruption of either complex not only reduces microtubule density but also microtubule bundling. These microtubule defects impair neurite formation, interfere with axon specification and growth, and disrupt axonal trafficking. In axons augmin does not merely mediate nucleation of microtubules but ensures their uniform plus end-out orientation. Thus, the augmin-γTuRC module, initially identified in mitotic cells, may be commonly used to generate and maintain microtubule configurations with specific polarity.

## Abstract (web summary)

In mature neurons the centrosome no longer functions as the main microtubule organizer and it is unclear how ordered microtubule arrays are assembled. Here, the authors show that in post-mitotic neurons this process depends on non-centrosomal nucleation mediated by the protein complex augmin and the nucleator gamma-TuRC.

## Graphical abstract

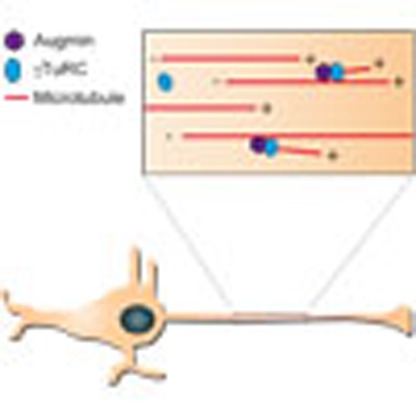

The neuronal microtubule cytoskeleton provides transport tracks for molecular cargos and organelles, and mediates essential processes such as neuron migration and polarization, neuritic growth and branching, and synaptic transmission^1^,^2^,^3^. Microtubules are polymers assembled from α-β-tubulin heterodimers and have an intrinsic polarity based on the head-to-tail arrangement of α- and β-tubulin. Neuronal microtubules appear predominantly bundled, presenting both parallel and anti-parallel configurations. Whereas in axons most of the microtubules are oriented with their plus ends away from the soma, microtubules in dendrites show mixed polarity, with a large fraction of microtubule plus ends oriented towards the soma. This specific organization of the microtubule cytoskeleton underlies the characteristic morphology and compartmentalization of neurons^1^,^3^,^4^,^5^.

Interestingly, most of the microtubules in mature neurons are not connected to the centrosome, the main microtubule organizing centre (MTOC) in many other cell types, raising the question of how non-centrosomal microtubules in neurons are nucleated and correctly positioned^4^. Early work established a model in which microtubules are nucleated at the centrosome, released, and transported into axons and dendrites by motor-dependent sliding along existing microtubules^6^,^7^,^8^,^9^,^10^,^11^,^12^. However, experimental removal of the centrosome affected neither axon growth in rodent cultured hippocampal neurons^13^ nor neuronal microtubule organization and morphogenesis in flies^1^,^2^,^3^,^14^,^15^. These results led to the conclusion that microtubules in post-mitotic neurons can be nucleated by a non-centrosomal mechanism. Together with non-centrosomal nucleation, severing of existing microtubules by katanin and spastin has also been proposed to generate new microtubules at non-centrosomal sites^1^,^3^,^4^,^5^,^16^,^17^. However, it is unclear how the polarity of locally generated microtubules would be controlled.

An essential component of all MTOCs is the protein γ-tubulin. γ-Tubulin, together with γ-tubulin complex proteins (GCPs), assembles into large γ-tubulin ring complexes (γTuRCs) that function as microtubule nucleators^4^,^18^. During neuron maturation γ-tubulin is gradually lost from centrosomes, correlating with a progressive loss of centrosomal nucleation activity^6^,^7^,^8^,^9^,^10^,^11^,^12^,^13^,^15^,^19^, but remains present in the cytoplasm. Non-centrosomal γ-tubulin was recently proposed to nucleate microtubules from dendritic Golgi outposts^13^,^20^ and from diffusible sites in the somato-dendritic compartment^21^. However, the existence of nucleation sites at dendritic Golgi outposts has subsequently been questioned^22^,^23^, and the mechanism by which γ-tubulin dependent, non-centrosomal nucleation occurs remains obscure.

In addition to nucleation at MTOCs, microtubules can also be nucleated from the lateral surface of pre-existing or ‘mother' microtubules. Such a mechanism generates microtubules within mitotic and meiotic spindles and within the interphase cortical microtubule array in plants, independently of centrosomes^24^. This nucleation mode requires another multi-subunit protein complex termed augmin, which recruits γTuRC to microtubule lattices to nucleate microtubule branches^25^,^26^,^27^,^28^,^29^.

Here, we demonstrate that augmin and γTuRC are crucial for microtubule organization in post-mitotic neurons. Non-centrosomal, augmin-γTuRC-dependent nucleation generates the highly bundled neuronal microtubule network and ensures uniform plus end-out microtubule polarity in axons. These functions are crucial for neuron morphogenesis and intracellular transport. Our results reveal the versatility of the augmin-γTuRC module and suggest that mature neurons may not require any specific MTOC to maintain the organization of their extensive microtubule network.

## Results

### γTuRC is present in neurons throughout development

**Figure 1:**
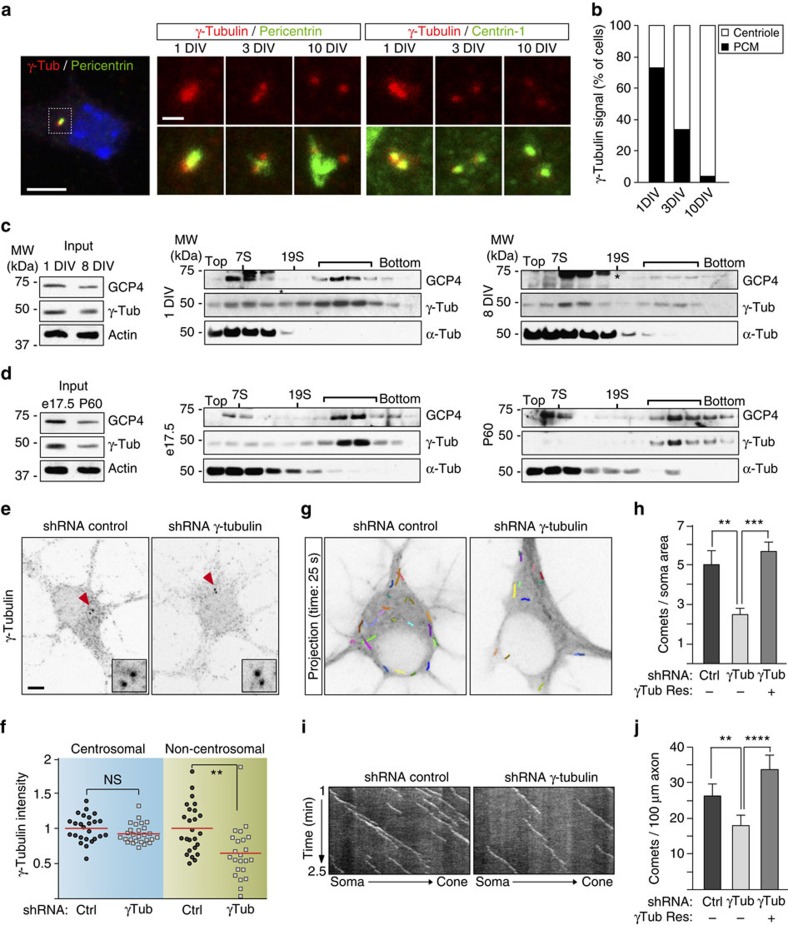
Non-centrosomal γTuRC is required for microtubule nucleation in somas and axons of hippocampal neurons. (**a**) Immunofluorescence images of centrosomes of neurons at the indicated DIV stained with the indicated antibodies. DAPI was used to stain nuclei. Scale bars, 5 μm and 1 μm (magnification). (**b**) Quantification of the distribution of centrosomal γ-tubulin in neurons shown in **a**. *n*=33 (1 DIV), 36 (3 DIV) and 25 (10 DIV) neurons, 3 independent cultures. (**c**) Lysates of cortical cultures at 1 or 8 DIV were fractionated on sucrose gradients. Input protein levels are shown, actin was used as loading control. The fractions were immunoblotted with the indicated antibodies. The asterisk labels an unspecific band recognized by the GCP4 antibody. The brackets mark γTuRC peak fractions. Aldolase (158 kDa, 7S) and thyroglobulin (669 kDa, 19S) were fractioned and used as molecular weight standards. Results were replicated twice. (**d**) Homogenates from hippocampi dissected at stage e17.5 or post-natal day 60 were analyzed on sucrose gradients as in **c**. (**e**) Representative immunofluorescence images of control and γ-tubulin-depleted neurons (transfected with shRNA #1, Fig. 4a) stained with γ-tubulin antibody. Red arrowheads mark centrioles. Scale bar, 5 μm. (**f**) Quantification of mean γ-tubulin immunofluorescence intensity at centrosomes and in the non-centrosomal cytoplasm. *n*=25 (control), 27 (depleted) neurons. Two independent experiments. **P<0.01, ns, not significant by the two-tailed *t*-test. Red bars show average. (**g**–**j**) Neurons were infected at 1 DIV with the indicated lentivirus, transfected at 4 DIV with the reporter EB3-Tomato and imaged by time-lapse microscopy 24 h later. (**g**) EB3-Tomato time-lapse projections with comet tracings of control and γ-tubulin-depleted somas. (**h**) Density of EB3-comets in the soma of control, γ-tubulin-depleted and γ-tubulin-depleted/rescued neurons. *n*=17, 15 and 21 neurons, respectively. Three independent experiments. **P<0.01, ***P<0.001 by one-way ANOVA followed by the Bonferroni's post hoc test. Error bars: s.e.m. (**i**) Kymographs of EB3-comets in control and γ-tubulin-depleted axons. (**j**) Quantification of comet density in control, γ-tubulin-depleted and γ-tubulin-depleted/rescued axons. *n*=38, 35 and 39 axons, respectively. Four independent experiments. **P<0.01, ****P<0.0001 in the Wald tests derived from a linear model. Error bars: s.e.m.

To address whether γTuRC may have a role in post-mitotic neurons we first determined the levels of γTuRC subunits at the centrosome of murine cultured hippocampal neurons and in the soluble fraction of hippocampal lysates. Previous work in rat hippocampal cultures suggested that despite the loss of centrosomal γ-tubulin, the cytosolic levels of γ-tubulin only moderately decreased during maturation. However, fully mature neurons were not analyzed^13^. We observed that at early stages of differentiation the γ-tubulin signal at centrosomes colocalized largely with the pericentriolar material marker pericentrin. During neuron maturation the centrosomal γ-tubulin signal progressively decreased, supporting previous findings^13^, but remained detectable even in the more mature stages (Fig. 1a,b; Supplementary Fig. 1a,b). Interestingly, we also found that contrary to early stages, the residual centrosomal γ-tubulin in more mature neurons was not co-distributed with the pericentriolar material, but was restricted to the centrioles (labelled by centrin antibody) (Fig. 1a,b). Thus, residual centrosomal γ-tubulin in mature neurons may represent a more stably bound, centriolar fraction of γ-tubulin^30^,^31^. A very similar decrease and redistribution was observed for the γTuRC targeting subunit NEDD1 (Supplementary Fig. 1a,b).

To determine the levels of cytosolic γTuRC we probed soluble extracts from cultured neurons and from hippocampal tissue dissected at different developmental stages by western blotting. After an initial, slight downregulation, the cytosolic levels of the γTuRC subunits γ-tubulin and GCP3 plateaued and remained constant throughout neuronal maturation in both experimental models. In contrast NEDD1, which targets γTuRC to centrosomes in proliferating cells^32^,^33^, was strongly downregulated and barely detectable in mature stages (Supplementary Fig. 1c,d).

To test whether cytosolic γ-tubulin in neurons assembled into γTuRC we used sucrose gradient centrifugation to size-fractionate extracts from cultured neurons or from hippocampal tissue at different stages of maturation. Indeed, in all cases some of the cytosolic γ-tubulin cofractionated with GCP4 at a molecular weight corresponding to the size of γTuRC (Fig. 1c,d). We conclude that, similar to proliferating cells, γ-tubulin in post-mitotic neurons assembles into γTuRC.[Fig f2][Fig f3]

### γTuRC nucleates microtubules in all neuronal compartments

To study the function of γ-tubulin in murine hippocampal neurons we depleted both forms of endogenous γ-tubulin (TUBG1 and TUBG2) by lentivirus-mediated short hairpin RNA (shRNA) transduction (Figs 1e,f and 4a, shRNA #1; Supplementary Fig. 2a). Expression of shRNA depleted mainly the cytosolic fraction of γ-tubulin since the residual γ-tubulin signal at centrosomes of 5 days *in vitro* (DIV) neurons was not further reduced (Fig. 1e,f), consistent with our earlier notion that the remaining centriolar γ-tubulin may turn over more slowly (Fig. 1a,b). Depletion of γ-tubulin was previously suggested to reduce microtubule nucleation in the soma and in dendrites (assayed by scoring EB3-labelled plus ends in fixed cells), thereby impairing dendritic arborization^21^. We used time-lapse imaging in lentivirus-transduced neurons transiently expressing EB3-Tomato and confirmed that under our experimental conditions depletion of γ-tubulin reduced the density of EB3-comets in the soma (Fig. 1g,h; Supplementary Movie 1). In addition, we found that γ-tubulin knock-down also reduced comet density in axons (Fig. 1i,j; Supplementary Movie 2), without affecting the average speed or distance covered by the comets (Supplementary Fig. 2b,c). In both somas and axons the reduction in comet density was fully rescued by co-expression of shRNA-resistant TUBG1 indicating the specificity of the phenotypes (Fig. 1h,j). Together with previous findings^21^,^22^ these results indicate that non-centrosomal γ-tubulin is required for microtubule nucleation in all compartments of mammalian neurons.

Surprisingly, in cells expressing exogenous TUBG1 we consistently observed a slight increase in the density of axonal EB3-comets compared with the control cells (Supplementary Fig. 2d). Moreover, whereas in control cells almost all axonal microtubules grew with their plus ends away from the soma, increased nucleation activity in cells expressing exogenous TUBG1 correlated with a small increase in the percentage of microtubule plus ends growing towards the soma (Supplementary Fig. 2e–g). These observations not only support the notion that γ-tubulin-dependent nucleation takes place in the axon but additionally suggest that regulation of microtubule nucleation and polarity may be mechanistically linked.

### Microtubules in γ-tubulin-depleted neurons are disorganized

**Figure 2 f2:**
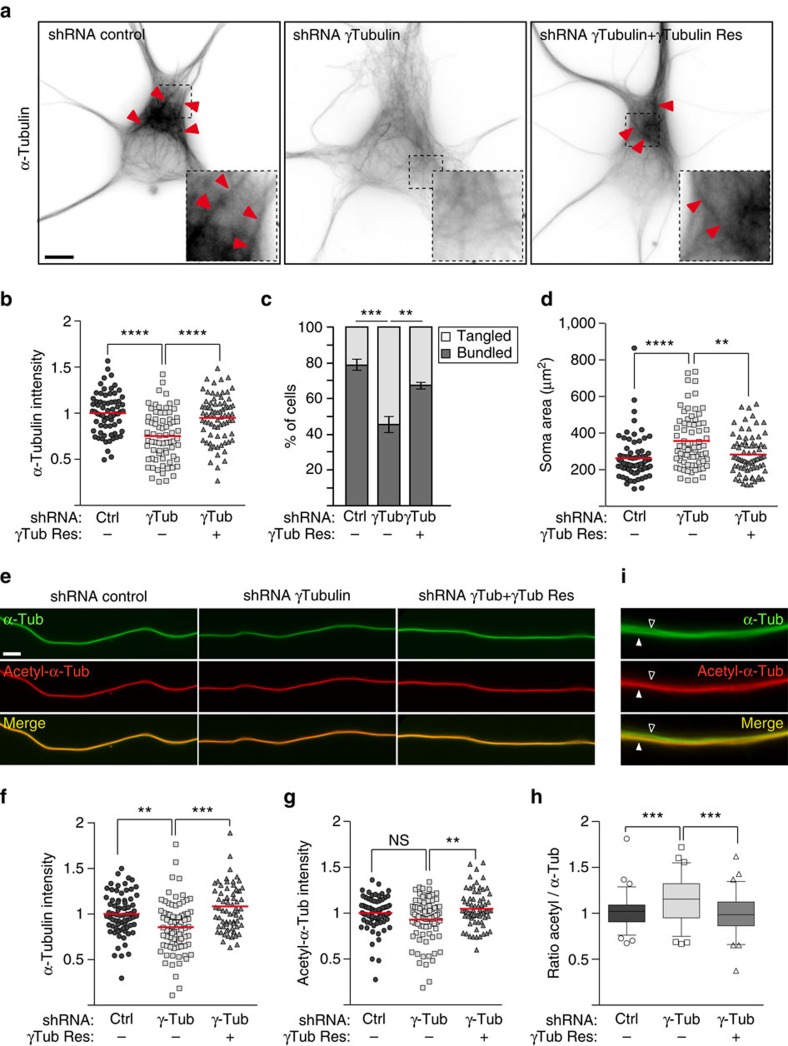
γ-Tubulin is essential to the density and organization of the microtubule network in somas and axons of hippocampal neurons. (**a**–**h**) Cultures were infected with lentivirus at 1 DIV, fixed at 5 DIV, and stained with the indicated antibodies. (**a**) Representative images of microtubules stained with α-tubulin antibodies in control, γ-tubulin-depleted and γ-tubulin-depleted/rescued neurons. Red arrowheads mark microtubule bundles in the soma. The areas in dashed squares are shown magnified for a better visualization of microtubule configurations. Scale bar, 5 μm. (**b**) Quantification of the mean intensity of α-tubulin staining in the somas of cells as in **a**. Values are normalized to the average intensity of axons in control cells. Red bars show average. (**c**) Quantification of neurons as in **a** containing tangled versus bundled microtubules. Error bars: s.e.m. (**d**) Quantification of the area covered by somas of neurons as in **a**. (**b**–**d**) *n*=68 (control), 72 (depleted), 74 (rescued) somas, three independent experiments. **P<0.01, ***P<0.001 and ****P<0.0001 by one-way ANOVA followed by the Bonferroni's test. Red bars show average. (**e**) Examples of α-tubulin and acetylated-α-tubulin co-stainings in the distal region of axons of control, γ-tubulin-depleted and γ-tubulin-depleted/rescued neurons. Scale bar, 5 μm. (**f**) Quantification of the mean intensity of α-tubulin staining in axons. Normalization was done as in **b**. Red bars show average. (**g**) Quantification of the mean intensity of acetylated-α-tubulin staining in the same axonal regions quantified in **f**. Normalization was done as in **b**. Red bars show average. (**h**) Ratios of acetylated-α-tubulin and total α-tubulin intensities in axons. (**f**–**h**) *n*=73 (control), 80 (depleted), 69 (rescued) axons, three independent experiments. **P<0.01, ***P<0.001, ns: not significant by one-way ANOVA followed by the Bonferroni's test. Boxes show 25–75 percentiles, whiskers show 5–95 percentiles, black bars show median. (**i**) Costaining of α-tubulin and acetylated-α-tubulin in a hippocampal axon. The white arrowhead marks an acetylated microtubule bundle, the open arrowhead marks a non-acetylated microtubule bundle.

To determine whether reduced nucleation in γ-tubulin-depleted neurons affected the microtubule network we fixed and stained neurons with antibodies under conditions that extracted most of the soluble tubulin but preserved microtubules^34^. Compared with the control neurons somas of depleted neurons displayed a ∼22% reduction in the intensity of total microtubule staining (Fig. 2a,b). Moreover, whereas most control cells contained robust microtubule bundles in the periphery of the soma and entering the neuritic shafts, microtubules in γ-tubulin-depleted cells appeared disorganized, with fewer cells displaying microtubule bundles (Fig. 2a,c). In addition, we noticed that somas of depleted neurons expanded over a larger area than somas of control cells (Fig. 2a,d). All of these defects could be rescued by expression of shRNA-resistant TUBG1. Similar to somas, axons in γ-tubulin-depleted neurons also contained fewer microtubules (Fig. 2e,f). Microtubules in axons are particularly stable and during neuron maturation become heavily modified by post-translational modification including tubulin acetylation^35^. However, even in mature neurons acetylated microtubules coexist with non-acetylated microtubules (Fig. 2i)^36^. Staining with antibodies against acetylated tubulin indicated that γ-tubulin depletion did not cause a reduction in acetylated axonal microtubules, suggesting that disruption of γTuRC primarily diminished the less stable population of microtubules (Fig. 2e,g,h).

**Figure 3 f3:**
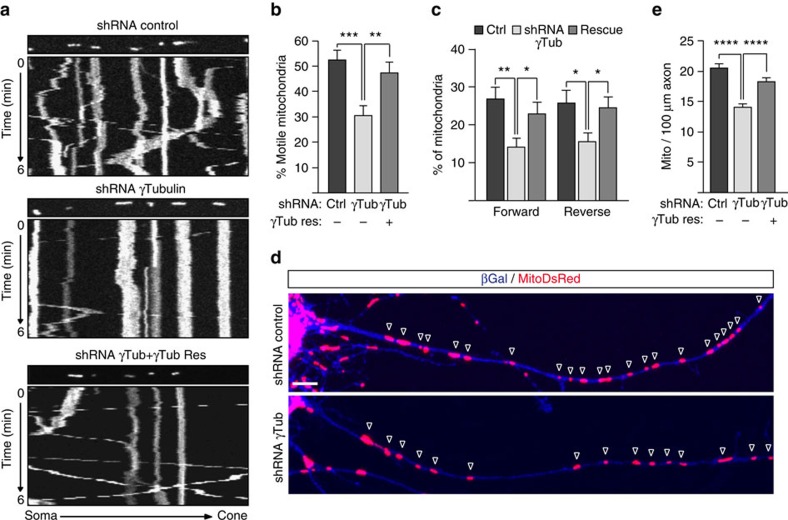
γ-Tubulin is required for mitochondrial transport in hippocampal axons. (**a**–**e**) Cultures were infected with the indicated lentivirus at 1 DIV, transfected at 4 DIV with the mitochondrial reporter MitoDsRed, and imaged 24 h later by time-lapse microscopy. (**a**) Representative kymographs displaying mitochondrial motility in control, γ-tubulin-depleted and γ-tubulin-depleted/rescued axons. The axonal region used for imaging is shown above the kymographs. (**b**) Quantification of the percentage of motile axonal mitochondria in different experimental conditions. (**c**) Quantification of the percentage of mitochondria moving forward and reverse along the axon in the different experimental conditions. (**b**,**c**) *n*=32 (control), 31 (depleted), 32 (rescued) axons, four independent experiments. *P<0.05, **P<0.01 and ***P<0.001 by one-way ANOVA followed by the Bonferroni's test. Error bars: s.e.m. (**d**) Representative images of the proximal region of the axon of transduced neurons co-transfected with MitoDsRed (Red) and β-galactosidase. (Blue) to visualize the axon. Open arrowheads mark the axonal mitochondria. Scale bar, 5 μm. (**e**) Quantification of the density of mitochondria within ∼100 μm proximal axon segments in control, γ-tubulin-depleted and γ-tubulin-depleted/rescued neurons. *n*=53 (control), 55 (depleted), 63 (rescued) axons, four independent experiments. ****P<0.0001 by one-way ANOVA followed by the Bonferroni's test. Error bars: s.e.m.

Due to the high density of microtubules in axons we could not discern qualitative changes in microtubule organization in this compartment. Instead we measured microtubule-based axonal transport as read-out for potential microtubule defects. Motility and distribution of mitochondria critically depend on microtubules^37^. Strikingly, depleting γ-tubulin reduced the proportion of motile mitochondria in hippocampal axons from ∼50 to ∼30%, a defect that was rescued by expression of RNAi-resistant wild-type γ-tubulin (Fig. 3a,b; Supplementary Movie 3). Analysis of the directionality of mitochondrial transport revealed that γ-tubulin depletion impaired similarly both anterograde and retrograde transport (Fig. 3c). We also observed changes in the steady state distribution of axonal mitochondria. Depletion of γ-tubulin reduced the density of mitochondria by ∼30% and this defect was rescued by shRNA-resistant TUBG1 (Fig. 3d,e). Together these results demonstrate that γ-tubulin has a critical role in maintaining microtubule organization and function in both soma and axon of hippocampal neurons.

### Morphogenesis of axons and dendrites requires γ-tubulin

**Figure 4:**
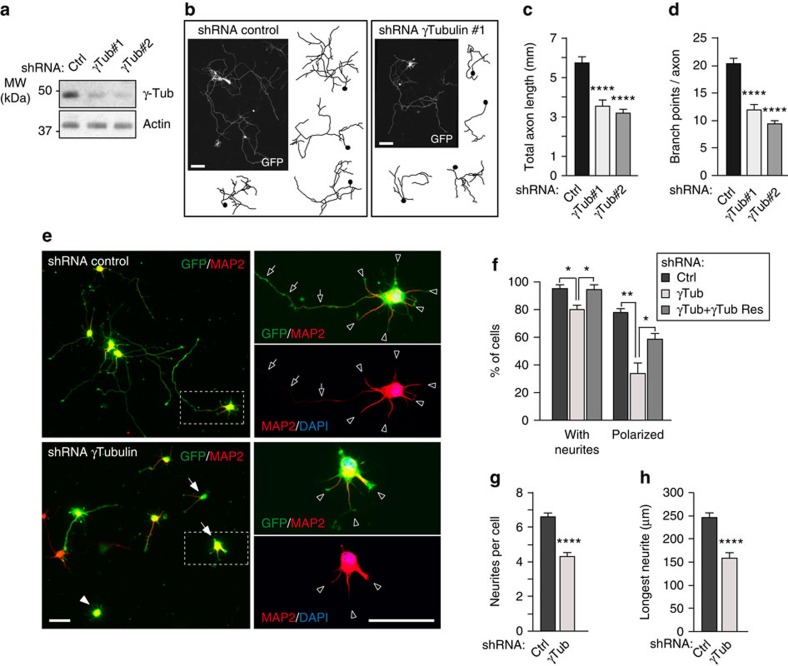
Depletion of γ-tubulin protein impairs neurite outgrowth as well as axon specification and arborization. (**a**) Western blot showing γ-tubulin protein depletion at 4 DIV using two different shRNAs. Actin protein was used as loading control. (**b**–**d**) Hippocampal neurons were transfected with plasmid expressing shRNA and GFP at 2 DIV, fixed and stained with GFP antibody at 7 DIV. (**b**) Representative images and drawings of GFP-stained control and γ-tubulin-depleted neurons. Scale bar, 100 μm. (**c**) Quantification total axon length of neurons as in **b**. (**d**) Quantification of the number of axonal branch points of neurons as in **b**. (**c**,**d**) *n*=42 (control), 39 (γTub#1), 42 (γTub#2) neurons, three independent experiments. ****P<0.0001 by two-tailed *t*-test. Error bars: s.e.m. (**e**–**h**) Hippocampal cultures were infected with the indicated lentivirus at 1 DIV, trypsinized, re-plated at 5 DIV and fixed at 6 DIV. (**e**) Representative images of GFP/MAP2 immunostainings of control and γ-tubulin-depleted re-plated cultures. White arrowheads mark neurons without neurites. White arrows mark neurons carrying neurites but lacking an axon-like process. Scale bar, 50 μm. The areas in dashed squares are presented magnified in the right part of the panel, with the indicated antibodies, to show morphological details of the neurons. Open arrows point to the MAP2-negative axon-like process. Open arrowheads mark the MAP2-positive neurites. (**f**) Quantification of the proportion of neurons extending one or more neurites and neurons showing at least one axon-like process in control, γ-tubulin-depleted and γ-tubulin-depleted/rescued neurons. *n*=153, 174 and 195 neurons, respectively, three independent replatings. *P<0.05, **P<0.01 by one-way ANOVA followed by the Bonferroni's test. Error bars: s.e.m. (**g**) Quantification of the average number of neurites extended by control and γ-tubulin-depleted neurons. *n*=152 (control), 174 (depleted), 178 (rescued) neurons, three independent replatings. (**h**) Measurement of the longest neurite in control and γ-tubulin-depleted neurons. (**g**,**h**) *n*=115 (control), 63 (depleted), 101 (rescued) neurons, three independent replatings. ****P<0.0001 by two-tailed *t*-test. Error bars: s.e.m.

Since microtubules are important determinants of neuron morphogenesis we examined whether loss of γ-tubulin affected axonal development in cultured hippocampal neurons. γ-Tubulin was depleted as before or with a different shRNA (shRNAs #1 and #2, respectively; Fig. 4a). In γ-tubulin-depleted 7 DIV neurons the total axon length was reduced by ∼40% compared with the control cells and the total number of axonal branch points was similarly reduced (Fig. 4b–d). To test whether loss of γ-tubulin also interfered with neurite formation and axon specification, neuronal cultures transduced with shRNAs for 4 days were trypsinized for a complete retraction of all processes and re-plated. After 24 h ∼95% of control neurons had initiated formation of MAP2-positive neurites and ∼80% had one MAP2-negative neurite with a thin and elongated, axon-like morphology (Fig. 4e,f). In contrast in γ-tubulin-depleted replatings, only ∼78% of neurons had MAP2-positive neurites and a minority (∼34%) appeared re-polarized with an axon-like process (Fig. 4e,f). In addition, the average number of neurites per cell and the length of the longest axon-like neurite were also reduced (Fig. 4e,g,h). Additional depletion experiments were performed in Neuro2A cells, a neuroblast cell line that can be stimulated with retinoic acid to promote cell cycle exit and neuritic outgrowth. Neuro2A cells transfected with plasmids expressing γ-tubulin shRNA (Supplementary Fig. 3a) and treated with retinoic acid formed fewer processes and total neurite length was reduced when compared with the control cells (Supplementary Fig. 3b–d). Together these results indicate that γ-tubulin is required for neurite outgrowth and extension as well as axon specification and arborization.

### γTuRC in post-mitotic neurons interacts with augmin

**Figure 5:**
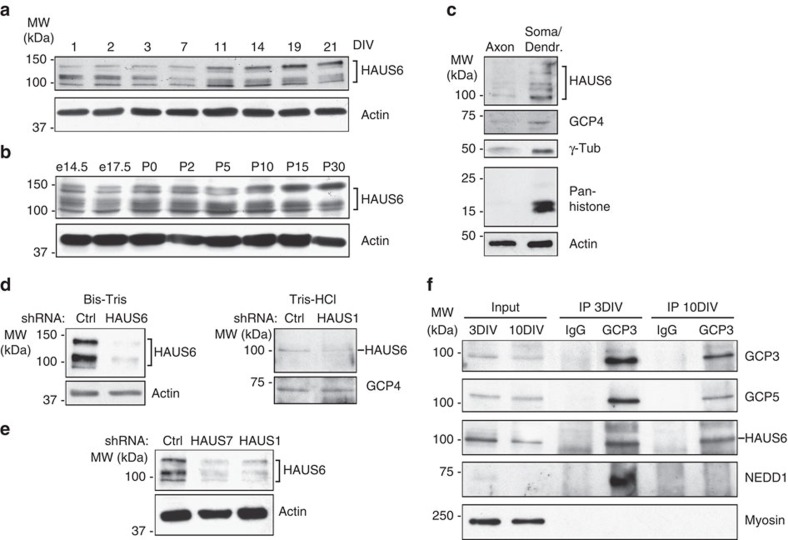
The augmin complex is present in the axon of post-mitotic neurons and interacts with γTuRC. (**a**,**b**) Western blot showing the levels of the augmin subunit HAUS6 in lysates from cultured neurons (**a**) and from hippocampal tissue (**b**) at different maturation stages. The antibody recognizes several bands in the 100–150 kDa range. Probing for actin was used as loading control. e, embryonic day; P, post-natal day. (**c**) Hippocampal neurons were cultured 8 DIV and protein homogenates of the axonal or somato-dendritic compartments were obtained separately. Equal protein amounts of each compartment were immunoblotted with the indicated antibodies. Pan-histone antibody was used as soma marker. Actin was used as loading control. This result was replicated twice. (**d**) Hippocampal cultures were infected with lentivirus at 1 DIV and analyzed by western blotting with HAUS6 antibody at 8 DIV (left) and 4 DIV (right). Two different gel systems were used: Bis-Tris gels (left) resolve several HAUS6 bands between 100 and 150 KDa (**a**–**c**,**e**) and Tris–HCl gels (right) resolve one major HAUS6 band at 100 KDa (**f**). Actin or GCP4 immunoblots were used as loading control. (**e**) Hippocampal cultures were infected with lentivirus at 1 DIV and analyzed at 4 DIV by western blotting with HAUS6 antibody. Probing for actin was used as loading controls. (**f**) Hippocampal cultures were lysed at 3 DIV or 10 DIV, and subjected to immunoprecipitation (IP) with control IgG or anti-GCP3 antibody. Immunoprecipitated proteins were detected by immunoblotting with antibodies against GCP3, GCP5, HAUS6, NEDD1 and myosin. This result was replicated three times. The protein in the inputs represents 2% of the total protein used for immunoprecipitation.

We sought to gain insight into the mechanism by which non-centrosomal γTuRC nucleated microtubules during neuron maturation. Since staining of rodent neurons with γ-tubulin antibodies did not reveal any structure with accumulation of γ-tubulin outside the centrosome (Fig. 1e)^13^,^21^, we speculated that post-mitotic neurons may generate new microtubules in an augmin-dependent manner, by nucleation from pre-existing microtubules rather than from a specific MTOC. Consistent with this possibility, western blotting of hippocampal cell lysate revealed that HAUS6, an augmin subunit implicated in binding to γTuRC^38^, is expressed in nerve cells throughout neuronal differentiation *in vitro* and *in vivo* (Fig. 5a,b). Probing of extracts prepared from separated neuronal compartments revealed that HAUS6 and GCP4 were present not only in the somato-dendritic compartment but also in axons, as previously shown for γ-tubulin^13^ (Fig. 5c). The identity of the detected HAUS6 protein was confirmed by shRNA-mediated depletion of augmin subunits (Fig. 5d). Depending on the gel system used for western blotting the HAUS6 antibody detected one or multiple bands, suggesting the presence of different HAUS6 isoforms or differential post-translational modification (Fig. 5d). Targeting independently two other augmin subunits, HAUS1 and HAUS7, by expression of shRNAs resulted in the co-depletion of HAUS6 (Fig. 5d,e). This interdependence of augmin subunits was observed previously in cycling cells^25^ and would be consistent with the presence of an augmin complex in post-mitotic neurons. Strikingly, despite the downregulation of the γTuRC adaptor NEDD1 (Supplementary Fig. 1), which mediates cooperation with augmin in other systems^24^,^32^,^38^,^39^, the augmin subunit HAUS6 could be co-immunoprecipitated with the γTuRC subunit GCP3 from lysates of both 3 DIV and 10 DIV neuronal cultures, indicating a physical interaction between both complexes (Fig. 5f). NEDD1 was detected in immunoprecipitates from young but not more mature cultures, suggesting that the interaction between γTuRC and augmin in mature neurons may be NEDD1-independent (Fig. 5f).

### Augmin depletion phenocopies the disruption of γTuRC

**Figure 6 f6:**
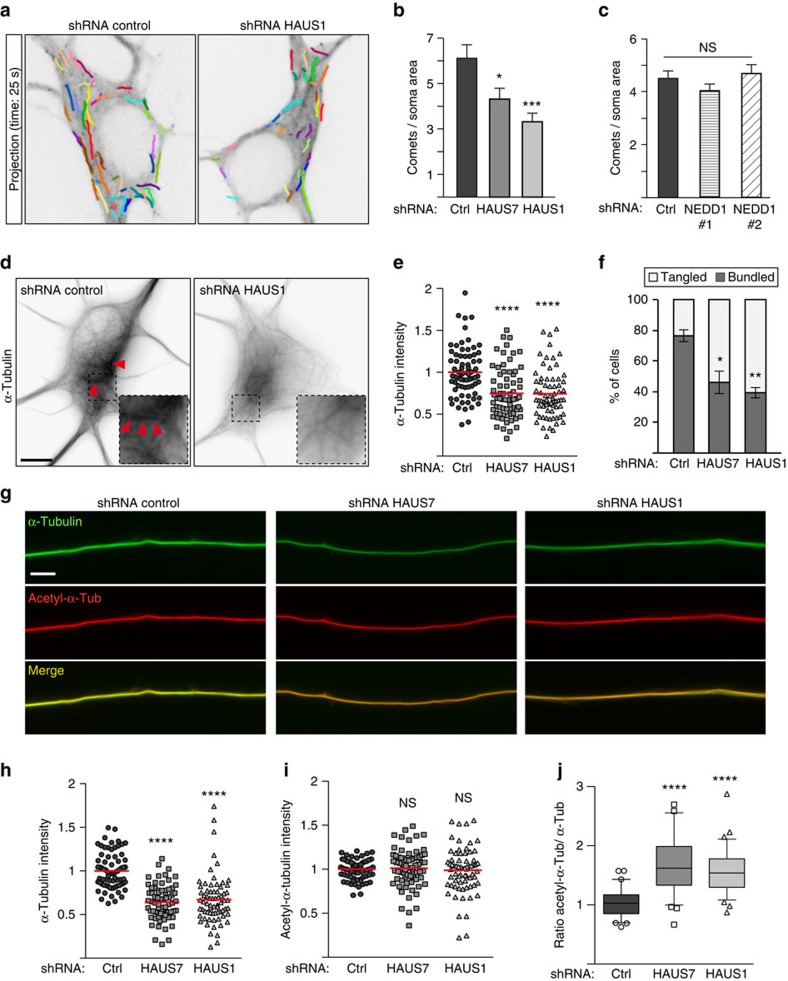
Augmin disruption phenocopies the defects observed in γ-tubulin-depleted neurons. (**a**–**c**) Hippocampal neurons were infected at 1 DIV with the indicated lentivirus, transfected with the reporter EB3-Tomato at 3 DIV (**a**,**b**) or 4 DIV (**c**), and imaged by time-lapse microscopy 24 h later. (**a**) Projection of frames from time-lapse recordings with tracings of EB3-comets. (**b**) Quantification EB3-comets density in control, HAUS7- and HAUS1-depleted neurons. *n*=25, 25 and 24 somas, respectively, three independent experiments. *P<0.05, ***P<0.001 by two-tailed *t*-test. Error bars: s.e.m. (**c**) Quantification EB3-comets density in control and NEDD1-depleted neurons. *n*=32 (control), 39 (NEDD1#1), 34 (NEDD1#2) somas, three independent experiments. ns: not significant by two-tailed *t*-test. Error bars: s.e.m. (**d**–**j**) Hippocampal cultures were infected with lentivirus as indicated at 1 DIV, simultaneously permeabilized and fixed at 4 DIV, and stained with the indicated antibodies. (**d**) Representative images showing microtubules stained with α-tubulin antibody in control and HAUS1-depleted neurons. Red arrowheads mark microtubule bundles in the soma. The areas in dashed squares are shown magnified for a better visualization of microtubule configurations. Scale bar, 5 μm. (**e**) Quantification of the mean intensity of α-tubulin staining normalized to the average intensity in control neurons. Red bars show average. (**f**) Scoring of neurons with bundled or tangled microtubules. (**e**,**f**) *n*=72 (control), 70 (HAUS7), 69 (HAUS1) somas, three independent experiments. *P<0.05, ***P<0.001, ****P<0.0001 by two-tailed *t*-test. Error bars: s.e.m. (**g**) Examples of α-tubulin and acetylated-α-tubulin co-stainings in axons of control, HAUS7 and HAUS1-depleted neurons. Scale bar, 5 μm. (**h**) Quantification of the mean intensity of the axonal α-tubulin signal normalized as in **e**. (**i**) Quantification of the mean intensity of the acetylated-α-tubulin signal in the same axon segments as in **h**. Values were normalized as in **e**. (**j**) Ratios of the intensities of acetylated-α-tubulin and total α-tubulin staining in axons. (**h**–**j**) *n*=72 (control), 69 (HAUS7), 69 (HAUS1) axons, three independent experiments. ****P<0.0001, ns: not significant by two-tailed *t*-test. Red bars in dot plots show average. Boxes show 25–75 percentiles, whiskers show 5–95 percentiles, black bars show median.

We next tested whether augmin had a role in neuronal microtubule nucleation. Indeed, depletion of either HAUS1 or HAUS7 caused a reduction in the density of EB3-labelled comets in the neuronal soma, similar to what we observed with γ-tubulin depletion (Figs 1g,h; 6a,b, [Fig f6]). In contrast, depletion of NEDD1 using two different shRNAs had no effect (Fig. 6c; Supplementary Fig. 4a). Staining of microtubules with α-tubulin antibodies revealed a reduction in both microtubule density and bundling in somas of augmin-depleted neurons (Fig. 6d–f), again similar to the defects caused by γ-tubulin depletion. The disruption of augmin also reduced total microtubule density in axons, without affecting acetylated axonal microtubules suggesting preferential loss of less stable microtubules (Fig. 6g–j). Indeed, the signal obtained with antibodies detecting tyrosinated tubulin, which is present in relatively young microtubules, was reduced in augmin-depleted axons (Supplementary Fig. 4b–d). In summary, the defects caused by depletion of augmin subunits were strikingly similar to the defects observed after depletion of γ-tubulin.

### Augmin controls microtubule polarity in axons

**Figure 7:**
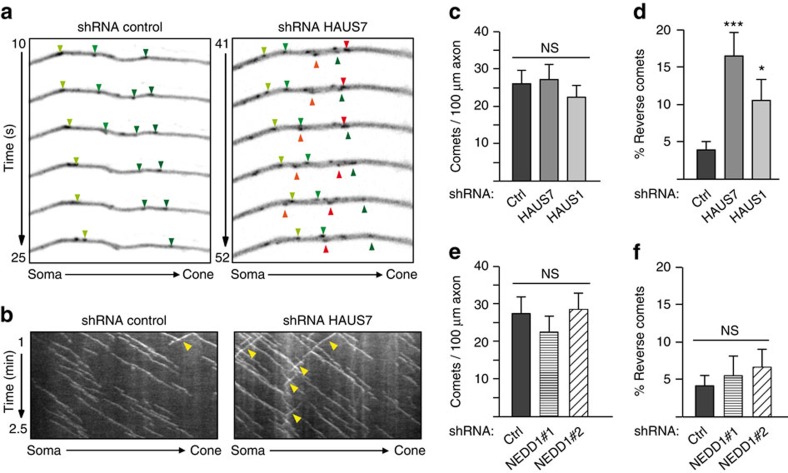
The polarity of axonal microtubules is regulated by the augmin complex. (**a**–**f**) Hippocampal cultures were infected with the indicated lentivirus at 1 DIV, transfected with the reporter EB3-Tomato at 3 DIV (**a**–**d**) or 4 DIV (**e**,**f**), and analyzed by time-lapse microscopy 24 h later. (**a**) Representative time-lapse frames of EB3-comets in control and HAUS7-depleted neurons. Green arrowheads mark comets moving away from the soma, red arrowheads comets moving in reverse. (**b**) Representative kymographs of time-lapse recordings of EB3-comets in control and HAUS7-depleted axons. Yellow arrowheads mark reverse comets. (**c**) Quantification of EB3-comet density in control and augmin-depleted axons. (**d**) Scoring of reverse comets in axons analyzed in **c**. *n*=30 (control), 25 (HAUS7), 27 (HAUS1) axons, three independent experiments. (**e**) Quantification of EB3-comet density in control and NEDD1-depleted axons. (**f**) Scoring of reverse comets in axons analyzed in **e**. *n*=33 (control), 32 (NEDD1#1), 34 (NEDD1#2), three independent experiments. (**c**,**e**) ns: not significant in the Wald tests derived from a linear model. Error bars: s.e.m. (**d**,**f**) *P<0.05, ***P<0.001 by two-tailed *t*-test. Error bars: s.e.m.

We hypothesized that augmin's capacity to generate microtubule arrays of uniform polarity^24^,^28^ may have important implications in axons in which almost all microtubules are oriented with their plus ends distal to the soma. To address this we infected hippocampal neurons with virus expressing shRNA directed against HAUS1 or HAUS7 and assessed microtubule orientation by quantifying the directionality of comets labelled by transiently expressed EB3-Tomato. Whereas in control neurons <4% of the growing plus ends of axonal microtubules were oriented towards the soma, in cells depleted of HAUS7 or HAUS1 this percentage increased to ∼17 and ∼11%, respectively (Fig. 7a,b,d; Supplementary Movie 4), while overall comet density was not changed (Fig. 7c). HAUS1 depletion also caused polarity defects in the axons of more mature neurons (10 DIV; Supplementary Fig. 5a–c) in which NEDD1 levels were naturally downregulated (Supplementary Fig. 1)^13^. This result supports our earlier notion that NEDD1 may not be required for augmin-dependent nucleation in post-mitotic neurons. Consistently, silencing of NEDD1 did not produce any changes in comet density or polarity in axons (Fig. 7e,f).

**Figure 8:**
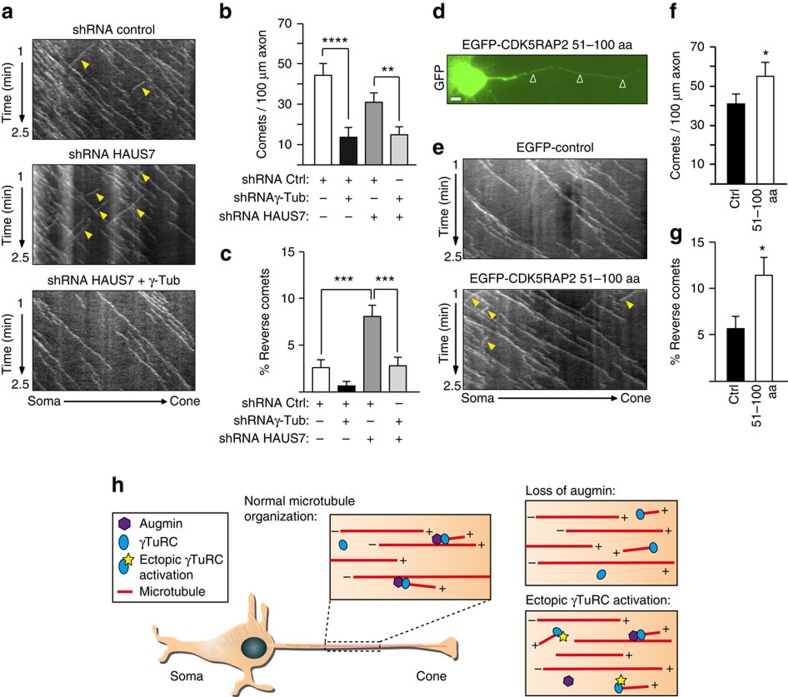
Augmin ensures the parallel orientation of axonal microtubules by restricting the nucleation of non-centrosomal γTuRC. (**a**–**c**) Neurons were infected consecutively with γ-tubulin and HAUS7 shRNA lentiviruses at 1 and 2 DIV, transfected with EB3-Tomato at 4 DIV, and imaged 24 h later. (**a**) Representative kymographs of EB3-comets in control, HAUS7-depleted and HAUS7/γ-tubulin double depleted axons. Yellow arrowheads point to reverse comets. (**b**) Quantification of EB3-comet density in control, γ-tubulin-depleted, HAUS7-depleted and HAUS7/γ-tubulin double depleted axons. (**c**) Scoring of reverse comets in axons analyzed in **b**. *n*=40 (control), 30 (γ-tubulin depleted), 46 (HAUS7 depleted), 48 (double depleted) axons, four independent experiments. (**d**–**g**) Hippocampal neurons were co-transfected at 3 DIV with EGFP or EGFP-tagged CDK5RAP2 fragment 51–100 together with EB3-Tomato. Imaging was performed at 4 DIV. (**d**) Immunofluorescence image of EGFP-tagged CDK5RAP2 51–100 in the axon. Scale bar, 5 μm. (**e**) Representative kymographs of EB3-comets in EGFP or EGFP-CDK5RAP2 51–100 transfected axons. Yellow arrowheads mark reverse comets. (**f**) Quantification of EB3-comet density in axons of EGFP (Ctrl) and EGFP-CDK5RAP2 51–100 (51–100) transfected neurons. (**g**) Scoring of reverse comets in the recordings analyzed in **f**. *n*=27 (control), 28 (51–100) axons, three independent experiments. (**b**,**f**) *P<0.05, **P<0.01, ****P<0.0001 in the Wald tests derived from a linear model. Error bars: s.e.m. (**c**,**g**) *P<0.05, ***P<0.001 by two-tailed *t*-test. Error bars: s.e.m. (**h**) Schematic representation illustrating microtubule nucleation and control of microtubule polarity in hippocampal axons, as well as defects observed upon either loss of augmin or ectopic γTuRC activation. Microtubule polarity is indicated by ‘plus' and ‘minus' symbols.

The increase in retrogradely growing axonal microtubules after augmin depletion could be interpreted to mean that augmin ensures nucleation of microtubules in parallel orientation along mother microtubules and that loss of augmin would cause ectopic and thus randomly oriented nucleation. If this model was correct, the co-depletion of γ-tubulin together with augmin should rescue the polarity defect. Indeed, no polarity defect was observed in neurons co-depleted of both HAUS7 and γ-tubulin (Fig. 8a–c). Another prediction of this model was that forcing ectopic nucleation by γTuRC may also increase the percentage of microtubules with reverse polarity, similar to augmin depletion. To test this we took advantage of the γTuRC-activating function of CDK5RAP2, a large scaffold protein found at the centrosome and the Golgi^40^. As predicted, overexpression of a small CDK5RAP2 fragment that harbours the γTuRC-activating region (CDK5RAP2 amino acids 51–100 or ‘γTuNA'^41^) increased not only the density of EB3-labelled comets in axons of hippocampal neurons but also the percentage of comets directed towards the soma (∼11% compared with the 5% in control cells; Fig. 8d–g; Supplementary Movie 5). We conclude that microtubules in axons of post-mitotic hippocampal neurons can be nucleated by non-centrosomal γTuRC, and that directional restriction of nucleation by augmin is required to correctly organize these microtubules with plus-end-distal orientation (Fig. 8h).

## Discussion

Generating the complex architecture of the mammalian brain requires precise control over the neuronal microtubule network to coordinate the distinct patterns of migration, neuritogenesis and maturation that characterize the heterogeneous neural cell population. This is demonstrated in part by the association of mutations in genes encoding tubulins, microtubule-associated proteins and molecular motors with a large spectrum of congenital brain malformations and neurodegenerative disorders^3^,^4^,^42^,^43^,^44^. For this reason, there has been a long-standing interest in deciphering how neuronal cells organize their extensive non-centrosomal microtubule arrays. Our work shows that mouse hippocampal neurons employ the augmin-γTuRC module to generate and organize microtubules. Previous work has implicated augmin-γTuRC dependent nucleation in the assembly of mitotic and meiotic spindles and of the plant-specific interphase cortical microtubule array^24^,^25^,^26^,^27^,^45^,^46^,^47^. On the basis of the results presented here we propose that this nucleation mechanism may be more common than previously anticipated.

Mutations in the genes encoding the γTuRC subunits γ-tubulin (*TUBG1*), GCP4 (*TUBGCP4*) and GCP6 (*TUBGCP6*) were recently identified in human patients suffering from brain malformations^42^,^48^,^49^,^50^, establishing γTuRC as an important player in brain development. In particular, in patients with mutations in *TUBG1*, cortical dysgenesis seems to be associated with migratory defects in developing neurons^42^. In coordination with their migratory path, neurons polarize and extend dendrites and axons^51^. In our study we observed that γ-tubulin deficiency impaired all of these processes and additionally disrupted axonal microtubule organization and transport. Even though mutations in genes encoding γTuRC subunits are known to cause centrosomal defects, it is tempting to speculate that impairment of the post-mitotic, non-centrosomal role of γTuRC that we describe here may contribute to the pathologies observed in patients with mutant γTuRC.

During neuron maturation, γ-tubulin is progressively lost from the centrosome, most likely due to downregulation of the targeting factor NEDD1 (this study)^13^. Interestingly, in the cortical microtubule array of interphase plant cells RNAi-mediated downregulation of NEDD1 reduces the angle at which daughter microtubules are nucleated along mother microtubules, biasing the balance towards a more parallel and bundled microtubule configuration^52^. Similarly, the developmental downregulation of NEDD1 in neurons may promote the bundled organization of microtubules in these cells. In mitotic systems, however, NEDD1 seems to have a role in mediating the cooperation between augmin and γTuRC^53^,^54^. Thus, one important issue to be addressed in future studies is how augmin and γTuRC in neurons cooperate in the absence of NEDD1. Another important question is whether augmin-γTuRC also stabilize the minus ends of nucleated microtubules or hand these over to CAMSAPs, which were recently shown to stabilize microtubule minus ends in neurons^21^,^55^,^56^,^57^. In this context it should be noted that branch structures have not yet been visualized in neuronal microtubule arrays. This may be explained by a high mobility of newly formed augmin-dependent microtubules, as observed in other systems^28^,^54^,^58^,^59^, which would cause branch structures to form only transiently. Indeed, even in the human mitotic spindle, which is composed of hundreds of microtubules, a recent 3D reconstruction by electron tomography revealed only very few microtubule minus ends that were visibly linked to the lattice of a ‘mother' microtubule^29^.

Work from several laboratories has demonstrated that centrosomal microtubule organization is not required for neuron differentiation and specification^13^,^14^,^15^. By implicating augmin in this process our study now provides a suitable mechanism to explain these results. However, centrosomal nucleation, release and transport of microtubules^7^ may coexist with augmin-dependent nucleation, in particular at early stages of neuronal differentiation, when the centrosome still retains some activity.

While augmin disruption largely phenocopied the microtubule defects caused by depletion of γ-tubulin, we also observed one important difference. Loss of augmin, but not of γ-tubulin, randomized microtubule polarity in the axon. This result reveals a key activity of augmin: controlling the orientation of newly nucleated microtubules. Interestingly, even though in axons augmin knockdown primarily affected the direction of nucleation rather than nucleation *per se*, the levels of axonal microtubules were lower than in controls. This suggests that microtubules nucleated in reverse orientation may be short-lived or subject to clearing^60^ and thus do not contribute to formation of the axonal microtubule array.

By promoting nucleation of daughter microtubules along mother microtubules, augmin may promote the formation of bundled microtubule configurations. This is supported by the loss of microtubule bundling and the appearance of tangled microtubules in both γTuRC and augmin-disrupted neurons. Independent of this nucleation-based effect, augmin may also promote bundling by direct interaction with microtubules^61^. Since previous studies in animal cells have shown that augmin, by nucleation of branches at relatively shallow angles, creates microtubule arrays of uniform polarity^28^,^29^, we have focused most of our analyses on axons. However, the ability of augmin to generate new microtubules based on the configuration of pre-existing microtubules, may also serve to organize the mixed polarity dendritic microtubule network. In this scenario the distinct microtubule configurations of axons and dendrites may be established by other mechanisms such as directed microtubule transport^11^,^12^, and the role of augmin-dependent nucleation would be reinforcement and maintenance of the compartment-specific arrays. Indeed, augmin has recently been implicated in microtubule formation in the dendrites of *Drosophila* sensory neurons^62^.

An increase in the number of axonal microtubule plus ends growing towards the soma has recently been described for gain- and loss-of-function alleles of γ-tubulin in *Drosophila*^22^. The provided evidence suggested a link between microtubule orientation and altered γTuRC nucleation activity. Based on our own results an alternative interpretation would be that the described γ-tubulin mutations may disrupt cooperation with augmin and/or induce ectopic nucleation. Our finding that ectopic nucleation can be induced by overexpression of a γTuRC activator implies that at least some γTuRC in axons is in a relatively inactive state. Deciphering how γTuRC may be activated on recruitment by augmin is an important goal for future studies.

In summary, the work presented here places augmin and γTuRC at the centre of microtubule organization in neurons. These two components together achieve what may be difficult to accomplish by MTOCs that are associated with specific cellular structures: assembling and maintaining microtubule arrays with specific polarity throughout an extensive, highly compartmentalized cytoplasm.

## Methods

### Animals and cell cultures

Pregnant 6-weeks-old female mice (*Mus musculus*; strain OF1) were purchased from Charles River and maintained at the animal facilities of the Barcelona Science Park (PCB), in strict accordance with the Spanish and European Union regulations. Work protocols have been approved by the Animal Care and Use Committee of the PCB (IACUC; CEEA-PCB), in accordance with applicable legislation. Hippocampal and cortical cell cultures^63^ were prepared from e17.5-18-5 mouse embryos. Briefly, tissue was dissected, treated with 0.25% trypsin (Life Technologies) during 15 min at 37 °C and dissociated into single cells by gentle trituration. Neurons were seeded on glass coverslips or plastic plates coated with 0.1 mg ml^−1^ Poly-D-lysine (Sigma) at ∼10^5^ cells per cm^2^ or 2 × 10^4^ cells per cm^2^ for low-density cultures. Neurons were plated in DMEM containing 10% fetal bovine serum (FBS), penicillin/streptomycin (pen/strep), and 1-2 h later medium was replaced by neurobasal medium supplemented with 2% B27, pen/strep, 0.6% Glucose and Glutamax (all reagents from Life Technologies). Cytosine arabinoside (1 μM; Sigma) was added to cultures at 3 DIV and 1/3 of the medium was refreshed every 4–5 days. Cultures of low density were supplemented at 2 DIV with conditioned media from mature cultures. HEK293T and Neuro2A cell lines were grown in DMEM containing 10% FBS and pen/strep. All cells were kept at 37 °C in a humidified atmosphere containing 5% CO_2_.

### Plasmids

The target sequences for simultaneous depletion of both TUBG1 and TUBG2 (γ-Tub#1: CAAGGAGGACATCTTCAA; γ-Tub#2: GGTTCGAGTTCTGGAAACA), HAUS6 (GGAGCTGATTGACACTTTA), NEDD1 (NEDD1#1: GCAGACATGTGTCGATTTA; NEDD1#2: GTCTAACCAAGCAAGAAAT) and luciferase (CTTACGCTGAGTACTTCGA) were cloned for expression as shRNAs into pLL3.7 (Addgene plasmid #11795)^64^. Plasmid pLL3.7-shRNA γ-Tub#1 was cloned and kindly provided by Tim Stearns' laboratory (Stanford University, USA). Other lentiviral plasmids with shRNAs were obtained from Sigma (HAUS1: GCTGAACTTACCAAGAAAGTA; HAUS7: CCAGATGACCAGGATCTTCTA; scrambled: CAACAAGATGAAGAGCACCAA). For rescue experiments, the cDNA of human γ-tubulin-MycHis^54^ was rendered shRNA resistant by PCR mutagenesis and cloned into pLL3.7-shRNA γ-Tub#1 and pLL3.7-luciferase-shRNA plasmids using NheI/EcoRI sites, replacing GFP. PCR was used to amplify a CDK5RAP2 fragment (amino acids 51–100) from the cDNA clone KIAA1633 (Kazusa DNA Research Institut, Kisarazu, Japan). The CDK5RAP2 51–100 sequence was inserted into pEGFP-C1 (Clontech). To generate bacterial expression plasmids for the production of His-tagged fragments of GCP3 and GCP5 the cDNA sequences corresponding to the N-terminal fragments of human GCP3 (amino acids 1–552), GCP4 (amino acids 1–347) and GCP5 (amino acids 1–713) were cloned into pET28a. The reporter plasmid EB3-Tomato was a generous gift of Anne Straube (University of Warwick, UK), and the mitochondrial reporter MitoDsRed was kindly provided by Antonio Zorzano (IRB Barcelona, Spain).

### Antibodies

To generate anti-GCP3, anti-GCP4 and anti-GCP5 antibodies, His-tagged N-terminal fragments of GCP3, GCP4 and GCP5 were expressed in ArcticExpress cells (Agilent), solubilized in 8 M urea and affinity-purified under denaturing conditions using Ni-Sepahrose beads (GE Healthcare) according to the manufacturer's protocol. The proteins were then used for immunization of rabbits (Antibody Production Service, Facultat de Farmacia, Universitat de Barcelona, Spain). GCP3, GCP4 and GCP5-specific antibodies were affinity-purified using the antigens subjected to PAGE and blotted onto membranes. The specificity of the antibodies was assayed by western blotting of Hela cell extract treated for 72 h with control siRNA or siRNA depleting GCP3 (GGACTTGCUAAAACCAGAA; Ambion), GCP4 (GCAATCAAGTGGCGCCTAA^41^) or GCP5 (GGAACATCATGTGGTCCATCA^65^) (Supplementary Fig. 6). Dilutions for western blot were 1:2,000 for anti-GCP3 and 1:1,000 for anti-GCP4 and anti-GCP5.

Other rabbit antibodies used in this study were: anti-NEDD1 (gift from S. Kumar, University of South Australia, Adelaide, Australia; dilution WB: 1:200; dilution IF: 1:500), anti-Pericentrin^32^ (dilution IF: 1:500), anti-HAUS6 (ref. 54) (dilution WB: 1:2,000), anti-Centrin3 (gift from A. Groen/R. Ori, dilution IF: 1:500), anti-GCP3 (Proteintech, dilution WB: 1:1,000), anti-MAP2 (Chemicon, dilution IF: 1:500) and anti-Myosin IIB heavy chain (Abcam #24761, dilution WB: 1:5,000). The mouse antibodies used were: anti-γ-tubulin (GTU-88, Sigma, dilution WB: 1:10,000; and TU-30, ExBio, dilution IF: 1:500), anti-α-tubulin (DM1A, Sigma, dilution IF: 1:2,000), anti-Acetylated-α-tubulin (6-11B-1, Sigma, dilution IF: 1:50,000), anti-actin (C4, MP Biomedicals, dilution WB: 1:10,000), anti-Pericentrin (BD Transduction Laboratories, dilution IF: 1:500), anti-Histone (gift of Ferran Azorín, IRB Barcelona, dilution WB: 1:1,000), anti-Tau-1 (PC1C6, Chemicon, dilution IF: 1:1,000), anti-β-Galactosidase (Promega, dilution IF: 1:1,000). Chicken anti-GFP (Aves Labs, dilution IF: 1:1,000). Rat anti-Tyrosinated-α-Tubulin (YL1/2, Millipore, dilution 1:5,000).

### Lentivirus production and transduction

Lentivirus was generated using the LentiLox3.7 system^64^. Briefly, HEK293T cells were co-transfected with pLL3.7 and the packaging plasmids with calcium phosphate, 72 h later lentivirus particles in the medium were concentrated by ultracentrifugation at 27,000 r.p.m. during 2 h. Virus particles were resuspended in PBS, aliquoted and stored at −80 °C. Infectivity was assayed for GFP-carrying virus by infecting HEK293T cells with serial dilutions of concentrated lentivirus, and sorting of GFP-positive cells by FACS 72 h after infection. Neurons were infected at 1 DIV at multiplicity of infection 3, in the case of low-density cultures we used multiplicity of infection 6. The complete medium was replaced with fresh medium 16–18 h after infection. For γ-tubulin/augmin double depletion, neurons were first infected with lentivirus encoding γ-tubulin shRNA at 1 DIV, followed by infection at 2 DIV with lentivirus encoding HAUS7 shRNA. Alternatively, the HAUS7 shRNA was provided by co-transfection of plasmid together with the EB3-tomato reporter 48 h before imaging. Both methods gave similar results. 16–18 h after each infection the complete medium was replaced. Infection efficiencies were determined at 4 DIV by GFP immunofluorescence analysis.

### Cell culture treatments

Young hippocampal neurons, shRNA-transduced neurons or more mature hippocampal neurons were transfected at 2 DIV, 4 DIV or 8 DIV, respectively, using Lipofectamine 2,000 (Life Technologies) according to manufacturer's instructions. To perform replating experiments, shRNA-transduced cultures at 5 DIV were trypsinized (0.05% Trypsin-EDTA, Life Technologies) for 5 min, collected in neurobasal medium with 5% FBS, pelleted at 800 r.p.m. for 3 min, resuspended in conditioned media and re-plated on poly-D-lysine coated coverslips. After 24 h of regrowth, neurons were fixed and stained for microscope analysis. The mouse neuroblastoma Neuro2A cells were transfected with Lipofectamine 2,000 and 72 h later differentiated by addition of 40 nM of retinoic acid (Sigma) plus 0.1% fetal calf serum (FCS) over 24 h before fixation.

### Protein extraction and immunoprecipitation

Cell cultures and hippocampal tissue samples were homogenized in lysis buffer (50 mM Tris pH 7.4, 150 mM NaCl, 1 mM MgCl_2_, 1 mM EGTA, 10% glycerol and 1% Triton X-100) in the presence of protease inhibitors (Complete, Roche). Lysates were clarified by centrifugation at 16,000*g* for 10 min at 4 °C. To obtain somato-dendritic or axonal protein extracts, hippocampal neurons were cultured on filters with 3 μm pore size membranes (Neurite Outgrowth Assay Kit, Millipore). At 8 DIV cells were fixed in methanol at −20 °C for 5 min. To obtain axonal fractions, somas on the upper side of the device were first carefully removed with wet flattened cotton swabs following manufacturer's instructions. Axons at the bottom side of the membrane were extracted in a 200 μl drop of SDS sample buffer. To obtain somato-dendritic fractions, axons from the bottom part of the device were first removed by scraping. Then SDS sample buffer extracts were prepared from the upper side of the membrane. For immunoprecipitation, 3 DIV or 10 DIV hippocampal cultures were lysed and cleared by centrifugation as above. Equal protein amounts were immunoprecipitated with anti-GCP3 (3 μl) antibody overnight. Protein G-Sepharose beads (GE Healthcare) were added during 2 h. After washing with lysis buffer, the proteins were eluted by boiling in 1 volume of 2 × sample loading buffer. For Western blotting all samples were boiled in sample buffer for SDS–PAGE, then proteins were separated and transferred onto PVDF membranes (Millipore). Membranes were blocked with 5% milk in TBST (20 mM Tris, pH 7.5, 150 mM NaCl, 0.05% Tween20) for 1 h and probed overnight with primary antibodies diluted in TBST. All uncropped western blots can be found in Supplementary Fig. 7.

### Sucrose gradient centrifugation

Cortical culture lysates and hippocampal homogenates were analyzed on sucrose gradients^66^ by loading equal protein amounts in lysis buffer (50 mM HEPES, pH 7.5, 150 mM NaCl, 1 mM MgCl_2_, 1 mM EGTA, 0.5% NP-40, with protease inhibitors) on a 10–40% sucrose gradient and centrifuged for 4 h at 55,000 r.p.m. at 4 °C. Fractions were collected and analyzed by immunoblotting. Aldolase (158 kDa, 7S) and thyroglobulin (669 kDa, 19S) (GE Healthcare) were centrifuged in parallel under identical conditions, and were used as molecular weight standards.

### Immunofluorescence microscopy

Cultured neurons and cell lines were fixed in methanol at −20 °C for 5 min or in 4% PFA/4% sucrose diluted in PBS for 15 min at room temperature. For staining microtubules^34^ cells were simultaneously permeabilized and fixed using 4% paraformaldehyde (PFA)/4% sucrose/0.25% glutaraldehyde/0.1% Triton X-100 diluted in PHEM buffer (60 mM Pipes, 25 mM Hepes pH 7.4, 5 mM EGTA, 1 mM MgCl_2_). All fixed cells were permeabilized with 0.25% Triton X-100 in PBS for 5 min, blocked with 4% bovine serum albumin (BSA, Sigma) and incubated in 2% BSA overnight with the primary antibodies as detailed in the Antibodies section. Alexa350, Alexa488, Alexa568 or Alexa633-coupled secondary fluorescent antibodies (Life Technologies) were used at dilution 1:250. Nuclei were stained with DAPI. Different samples within one experiment were imaged using constant intensity and exposure settings, avoiding signal saturation. An inverted confocal microscope TCS-SP2 AOBS (Leica Microsystem, GmbH) equipped with a 63 × /1.40 OIL objective was used for imaging centrosomes and axonal mitochondria at 1,024 × 1,024 pixel resolution. Centrosome image stacks were taken with a 0.3–0.4 μm step-size, and mitochondria density in axons was analyzed in β-Gal/MitoDsRed-transfected neurons by imaging a ∼100 μm axon segment proximal to the soma. For analysis of microtubule stainings, single-plane images of neuron somas and isolated axon shafts within constant distance from the growth cone were acquired with an Orca AG camera (Hamamatsu) coupled to a Leica DMI6000B microscope equipped with a 100 × /1.40 OIL objective. 10 × /0.30 DRY, 20 × /0.50 DRY, and 40 × /1.25 OIL objectives were additionally used for standard imaging and for mosaics generation of complete axons and re-plated neurons.

### Time-lapse microscopy

Hippocampal cultures were plated in glass-bottom dishes (MatTek), transduced with virus expressing shRNA, transfected with either EB3-Tomato or Mito-DsRed reporters at 3/4 DIV and imaged 24 h later. For analysis after expression of EGFP-CDK5RAP2 51–100, cultures were co-transfected with plasmid encoding EGFP or fragment EGFP-CDK5RAP2 51–100 together with EB3-Tomato reporter (2:1 ratio) at 3 DIV and imaged 24 h later. For comet analysis in more mature neurons, cultures were co-transfected with shRNAs together with EB3-Tomato reporter (2:1 ratio) at 8 DIV and imaged 48 h later. Live-imaging of EB3-comets and of mitochondria was performed in the soma and/or within the proximal axons of random transfected cells, using an Olympus IX81 microscope equipped with Yokogawa CSU-X1 spinning disc and a temperature controlled CO_2_ incubation chamber. Image stacks were acquired with 100 × /1.4 OIL immersion objective and an iXon EMCCD Andor DU-897 camera, using iQ2 software. Fluorescent images with pixel size of 0.14 μm were taken at 1 s intervals during 2.5 min for EB3-comets in axons, at 0.5 s intervals during 25 s for EB3-comets in somas, and at 3 s intervals during 6 min for axonal mitochondria.

### Image analysis

All images were processed and quantified using the ImageJ software (NIH). For all fluorescence intensity measurements, background signal was measured in an adjacent area and subtracted. For centrosome quantifications, equal numbers of confocal planes were projected and the intensity within a fixed circular area around the centrosomes was measured in each cell. Soma EB3 comet density was obtained using an ImageJ custom-written macro and normalized to the neuronal soma area. Axonal EB3 comet and mitochondria analysis were performed using the kymograph macro (ImageJ software), with lines drawn on the trajectories of comets and mitochondria, respectively. The average fluorescence intensities of α-tubulin, acetylated α-tubulin, and tyrosinated α-tubulin stainings were measured within the soma area or along the imaged axonal trace.

For scoring cells with microtubule bundles we took advantage of the higher intensity of the α-tubulin signal in bundled compared with the tangled configurations. α-Tubulin images of the somas were thresholded (ImageJ) using the average of the auto threshold values of control cells in each experiment. The thresholded images highlighted features with high staining intensity. A binary mask was obtained from the thresholded image and overlayed with the 8-bit grey scale image of the stained microtubules for identification and scoring of microtubule bundles. High intensity microtubule signals that were not bundles were not scored. Cells were classified as ‘bundled', when showing at least one microtubule bundle in the soma, other cells were classified as ‘tangled'.

Whole axon and neurite length were measured using the NeuronJ macro (ImageJ software).

### Statistic analysis

Statistic analysis was done using the Prism 6 software. Two-tailed unpaired t-tests or ANOVA tests were performed to compare the experimental groups. The details are reported in the figures and the figure legends. For Figs 1j, 7c,e and 8b,f and Supplementary Figs 2d and 5b group means differences were assessed using a linear model including the experiment run as a covariate. As a deviation from the normal distribution was observed in the data, a transformation was applied to the data in order to meet the assumptions of the model. This transformation was chosen from the Box–Cox family as it showed to be optimal according to the Maximum Likelihood criteria^67^. For interpretation purposes, results are expressed as adjusted means and standard errors in their original scale after undoing this transformation. For doing so, standard errors were computed from 1,000 simulations generated by the corresponding model using the R package arm (http://CRAN.R-project.org/package=arm)^68^. In addition and for visualization purposes, adjusted means were calibrated to the overall comet density of Fig. 1j. A 5% level was chosen for significance of group differences after multiple contrasts adjustment^69^.

### Data availability

The authors declare that all the data supporting the findings of this study are available within the article and its Supplementary Information files, or available upon request from the authors.

## Additional information

**How to cite this article:** Sánchez-Huertas, C. *et al.* Non-centrosomal nucleation mediated by augmin organizes microtubules in post-mitotic neurons and controls axonal microtubule polarity. *Nat. Commun.* 7:12187 doi: 10.1038/ncomms12187 (2016).

## Supplementary Material

Supplementary Information Supplementary Figures 1-7

Supplementary Movie 1 (related to Fig. 1g, h) Depletion of γ-tubulin reduces microtubule nucleation in the soma of hippocampal neurons. Cultured neurons were infected with lentivirus carrying shRNAs and transfected at 4 DIV with the reporter EB3-Tomato during 24 hours before live-imaging analysis.

Supplementary Movie 2 (related to Fig. 1i, j) Reduction of γ-tubulin levels impairs microtubule nucleation in axons. Transduced hippocampal neurons were transfected with EB3-tomato reporter at 4 DIV and the axons were imaged by time-lapse microscopy. The soma of the neurons is located at the left side of the videos.

Supplementary Movie 3 (related to Fig. 3a, b) Mitochondrial motility in axons is impaired in γ-tubulin depleted neurons. Hippocampal cultures were infected with the indicated lentivirus, transfected with the mitochondrial reporter MitoDsRed at 4 DIV and the axons were imaged 24 hours later by time-lapse microscopy. The soma of the neurons is located at the left side of the videos.

Supplementary Movie 4 (related to Fig. 7a-d) The augmin complex is required for the uniform polarity of microtubules in hippocampal axons. Neurons were infected with the indicated lentivirus and transfected with EB3-Tomato 24 hours before imaging the axons by time-lapse microscopy. The soma of the neurons is located at the left side of the videos.

Supplementary Movie 5 (related to Fig. 8e-g) Overactivating γTuRC by expression of the 51-100 aa conserved region of CDK5RAP2 promotes mixed microtubule polarity in axons. Hippocampal neurons were co-transfected at 3 DIV with EGFP or EGFP-CDK5RAP2 51-100 together with the EB3-Tomato reporter and axons were imaged 24 hours later. The soma of the neurons is located at the left side of the videos.
