# Supplementary material for: Non-centrosomal nucleation mediated by augmin organizes microtubules in post-mitotic neurons and controls axonal microtubule polarity
Source: Nat Commun. 2016 Jul 13;7:12187. doi: 10.1038/ncomms12187 (PMC4947180; doi:10.1038/ncomms12187)
Supplement: Supplementary Information — Supplementary Figures 1-7 [file ncomms12187-s1.pdf]

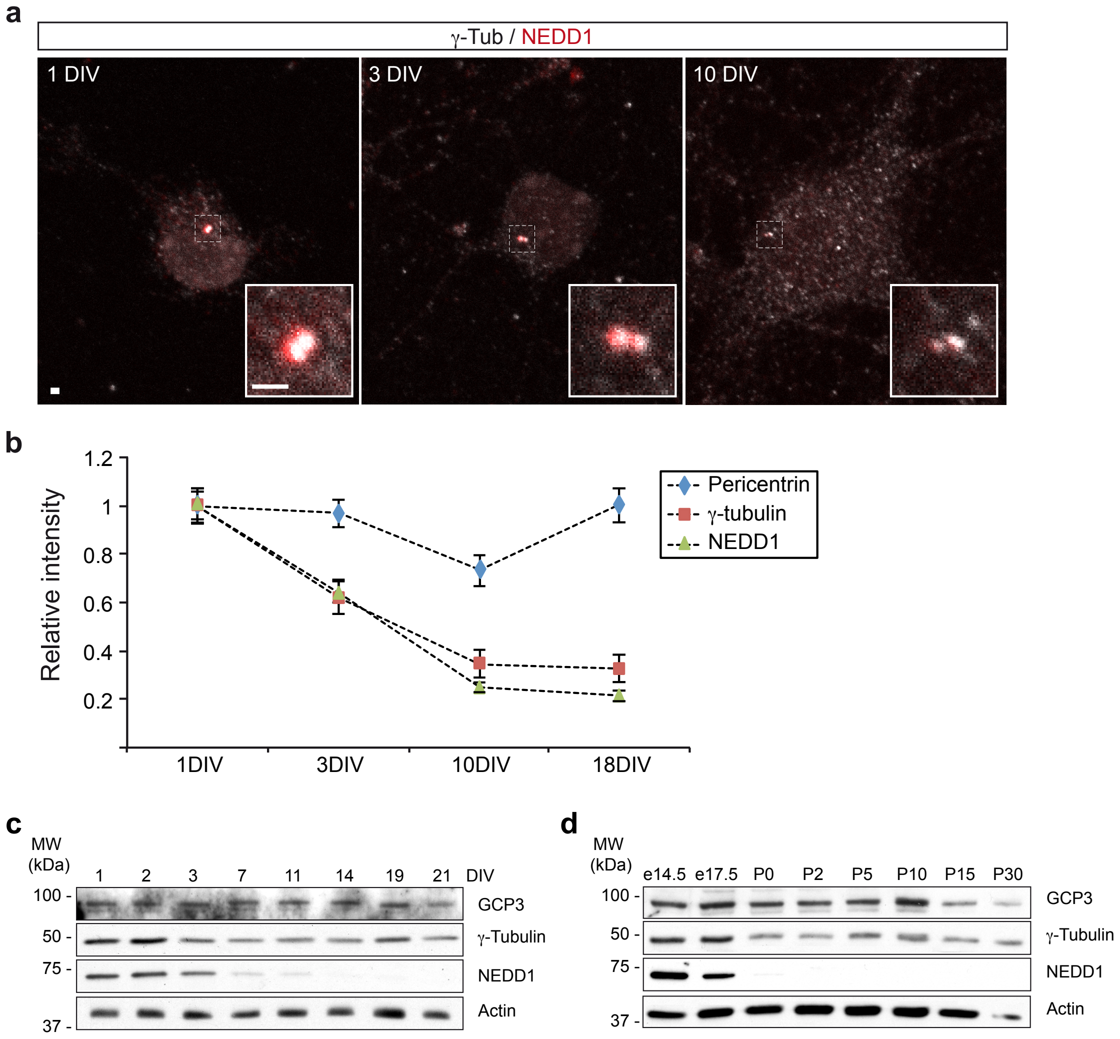

Supplementary Figure 1 The γTuRC subunits NEDD1 and γ-tubulin are depleted from centrosomes during hippocampal maturation, but total cytosolic levels remain constant. **(a)** Microscopy images of cultured hippocampal neurons immunostained with antibodies against γ-tubulin (white) and NEDD1 (red). Magnifications of the centrosomes (dashed squares) are shown. Scale bars, 1 μm. **(b)** Quantification of the mean intensity of the centrosomal signal of pericentrin, γ-tubulin and NEDD1 during the maturation process. n=32 (1DIV), 29 (3DIV), 30 (10DIV), 27 (18DIV) neurons, 3 independent cultures. Error bars show s.e.m. **(c, d)** Western blot showing the levels of the γTuRC subunits γ-tubulin, GCP3 and NEDD1 in lysates from cultured neurons **(c)** and from hippocampal tissue **(d)** at different maturation stages. Detection of actin was used as loading control. These results were replicated twice.

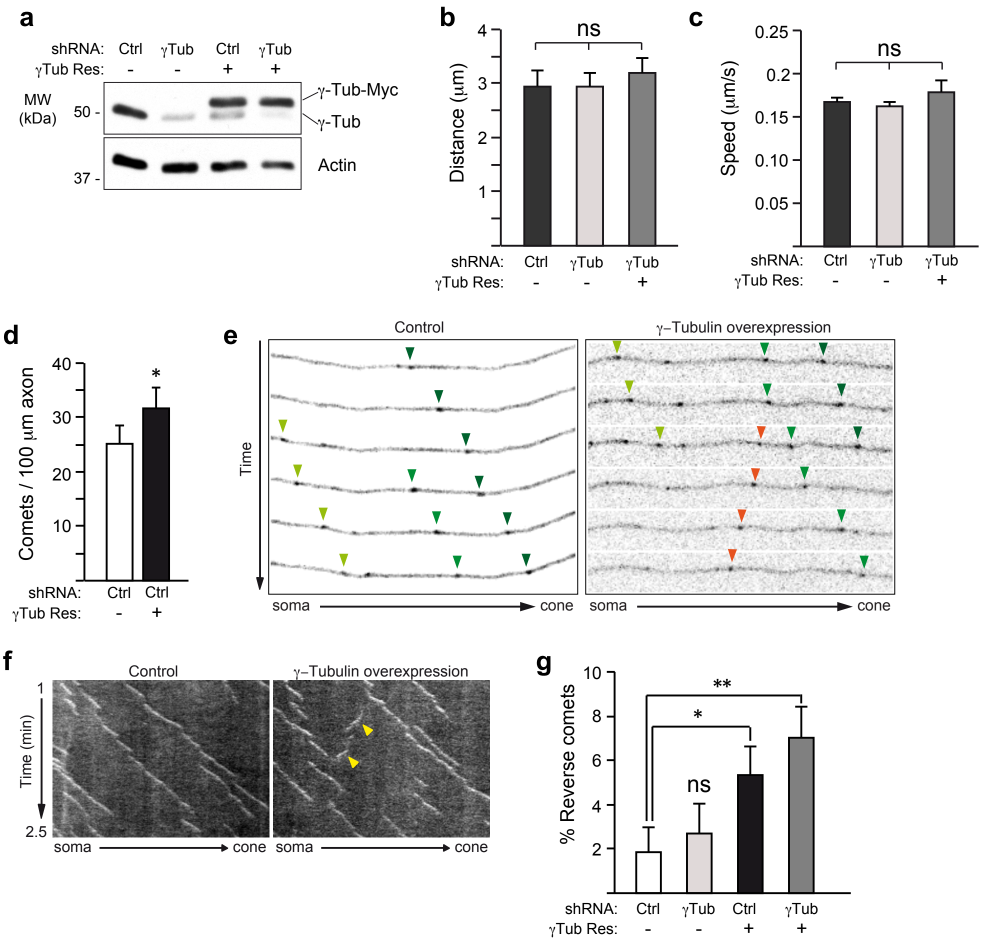

Supplementary Figure 2 γ-Tubulin depletion does not interfere with microtubule growth parameters in axons, but γ-tubulin overexpression stimulates nucleation and increases the percentage of retrograde microtubule growth. **(a)** Cultures were infected with lentivirus expressing control or γ-tubulin shRNA in combination with MycHis-tagged γ-tubulin. 7 days later lysates were probed by western blot with antibodies against γ-tubulin and actin as loading control. **(b-g)** Hippocampal neurons were infected at 1 DIV with the indicated lentivirus and transfected with the reporter EB3-Tomato at 4 DIV for 24 hours before imaging by time-lapse microscopy. **(b, c)** Quantification of the average distance **(b)** and speed **(c)** of EB3 comets in control, γ-tubulin depleted and γ-tubulin depleted/rescued axons. n=906 comets analyzed in 38 (control) axons, 677 comets analyzed in 35 (depleted) axons, 1149 comets analyzed in 39 (rescued) axons, from 4 experiments. ns, not significant by one-way ANOVA. Error bars: s.e.m. **(d)** Quantification of EB3 comet density in the axons of control and γ-tubulin overexpressing neurons. n=38 (control), 34 (γ-tubulin overexpression), 4 independent experiments. *P<0.05 in Wald tests derived from a linear model. Error bars: s.e.m. **(e, f)** Representative time-lapse frames **(e)** and kymographs **(f)** showing the dynamics of EB3-comets in control and γ-tubulin overexpressing axons. Arrowheads in shades of green mark comets moving towards the cone, arrowheads in shades of red and yellow mark comets moving in the opposite direction. **(g)** Quantification of the percentage of comets moving towards the soma in the recordings analysed in **d** and in **Figure 1j**. n=36 (control), 35, (depleted), 34 (γ-tubulin overexpression), 37 (rescued) axons, 4 experiments. *P<0.05, P<0.01, ns: not significant by two-tailed *t*-test. Error bars: s.e.m.

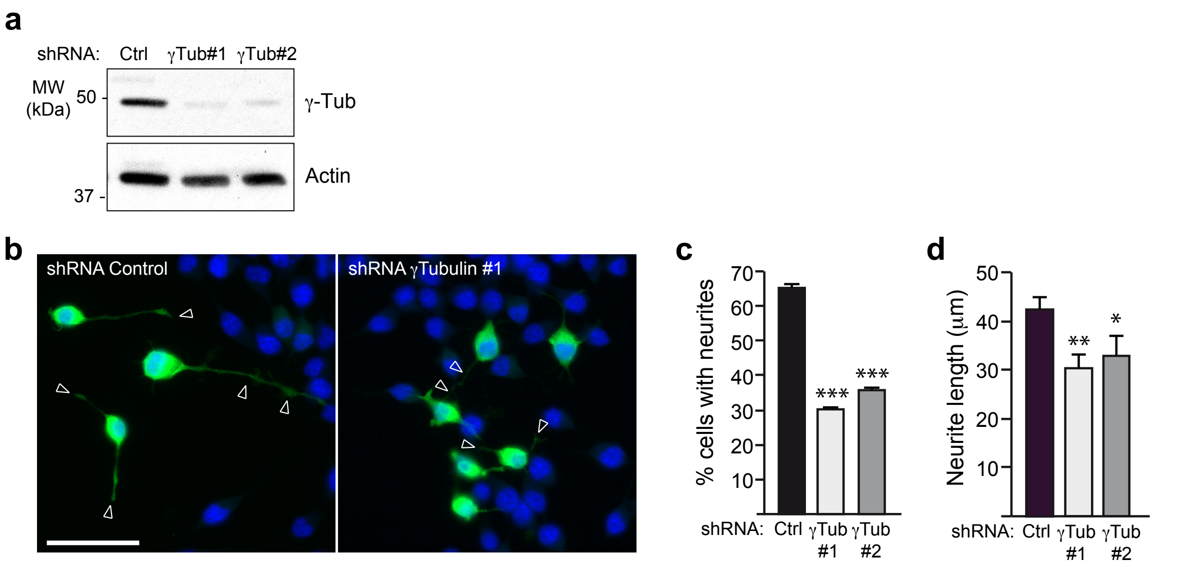

Supplementary Figure 3 Depletion of γ-tubulin impairs neurite outgrowth in the cell line Neuro2A. **(a)** Neuro2A cells were transfected with plasmids expressing shRNA and GFP during 72 hours. γ-tubulin protein levels were analysed by western blot. Actin protein was used as loading control. **(b-d)** Neuro2A cells were transfected as in **a**, differentiated with retinoic acid during 24 hours to promote neurite elongation and fixed for analysis. **(b)** Representative immunostainings of GFP in control and γ-tubulin depleted Neuro2a cells. DAPI was used to stain nuclei. Scale bar, 50 μm. Open arrowheads mark the neurites. **(c)** Quantification of the percentage of cells with at least one neurite. n=174 (control), 199 (γTub shRNA #1), 260 (γTub shRNA #2) cells, 3 independent experiments. **(d)** Measurement of the total neurite length per cell in control and γ-tubulin depleted Neuro2A cells. n=124 (control), 86 (γTub shRNA #1), 89 (γTub shRNA #2) cells, 3 independent experiments. *P<0.05, **P<0.01, ***P<0.001 by two-tailed *t*-test. Error bars show s.e.m.

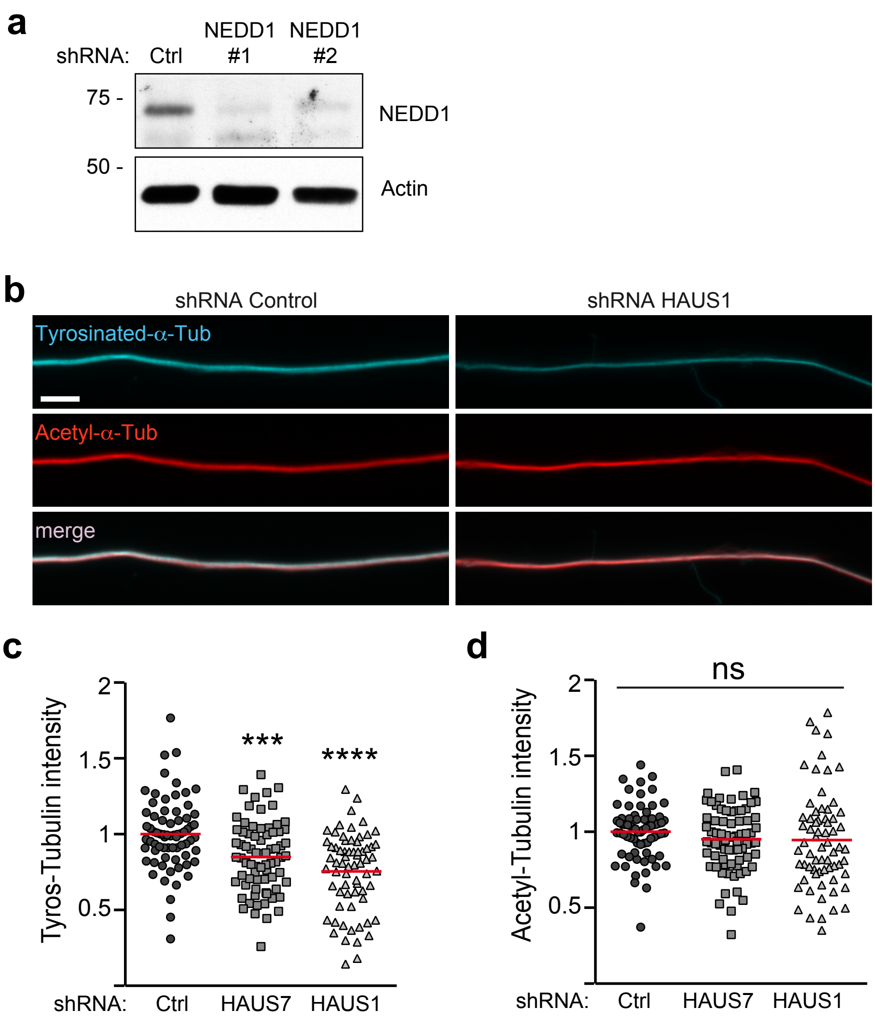

Supplementary Figure 4 Depletion of NEDD1 in hippocampal neurons and reduction of tyrosinated microtubules in augmin-depleted axons. **(a)** Hippocampal cultures were infected at 1 DIV with lentivirus and lysed at 5 DIV. Proteins were immunoblotted with the indicated antibodies. Actin was used as loading control. **(b-d)** Hippocampal cultures were infected with lentivirus at 1 DIV, simultaneously permeabilized and fixed at 4 DIV, and stained with the indicated antibodies. **(b)** Representative images showing co-staining of tyrosinated (cyan) and acetylated (red) α-tubulin in control and HAUS1-depleted neurons. **(c)** Quantification of the mean intensity of the axonal tyrosinated-α-tubulin signal normalized to the average intensity in control neurons. **(d)** Quantification of the mean intensity of the acetylated-α-tubulin signal in the same axon segments as in **c**. Values were normalized as in **c**. n=71 (control), 73 (HAUS7 shRNA), 67 (HAUS1 shRNA) neurons, 3 independent experiments. ***P<0.001, **** P<0.0001, ns: not significant by two-tailed *t*-test. Red bars in dot plots show average.

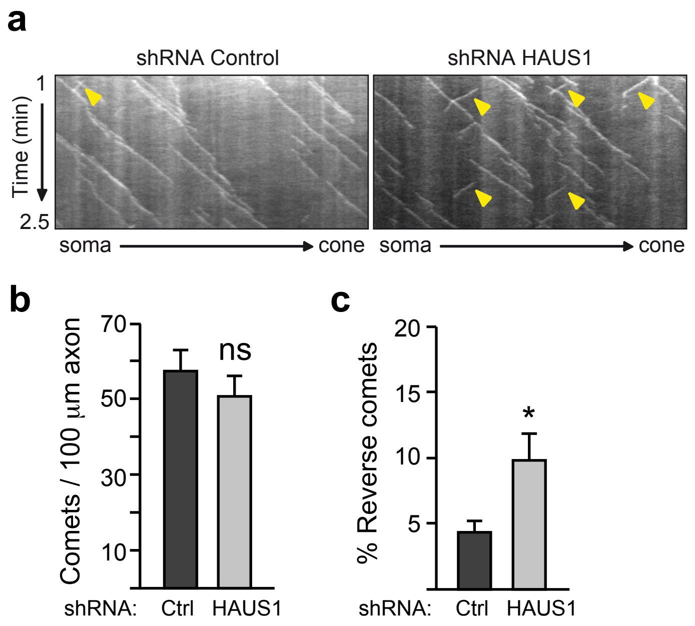

Supplementary Figure 5 Augmin is required for maintaining correct microtubule polarity in axons of mature neurons. **(a-c)** Hippocampal neurons were co-transfected at 8 DIV with plasmids expressing control or HAUS1 shRNA together with EB3-Tomato. Time-lapse imaging was performed at 10 DIV. **(a)** Representative kymographs of EB3-comets recorded in control and HAUS1-depleted axons. Yellow arrowheads point to comets moving towards the soma. **(b)** Quantification of EB3-comet density in axons of control and HAUS1-depleted neurons. **(c)** Scoring of comets moving towards the soma in the recordings analyzed in **b**. n=31 (control), 28 (HAUS1 shRNA) axons, 2 independent experiments. ns: not significant in Wald tests derived from a linear model. *P<0.05 by two-tailed *t*-test. Error bars show s.e.m.

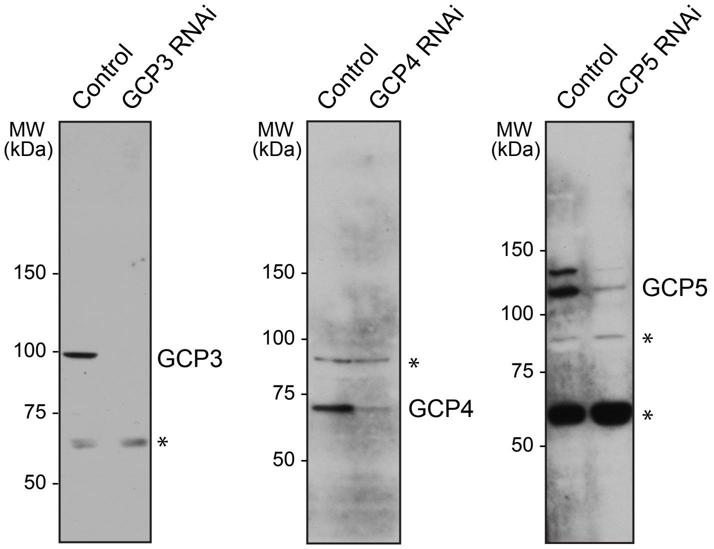

Supplementary Figure 6 Specificity test for antibodies raised against GCP3, GCP4, and GCP5. Purified antibodies were used to probe western blots of whole cell extract from Hela cells treated with control RNA or siRNA to deplete GCP3, GCP4, or GCP5, as indicated. Unspecific bands are labelled by asterisks and serve as loading control. In each case the antibody recognizes a band that is of the expected size and is diminished after RNAi. The specific bands are labelled by the protein names.

Figure 1c

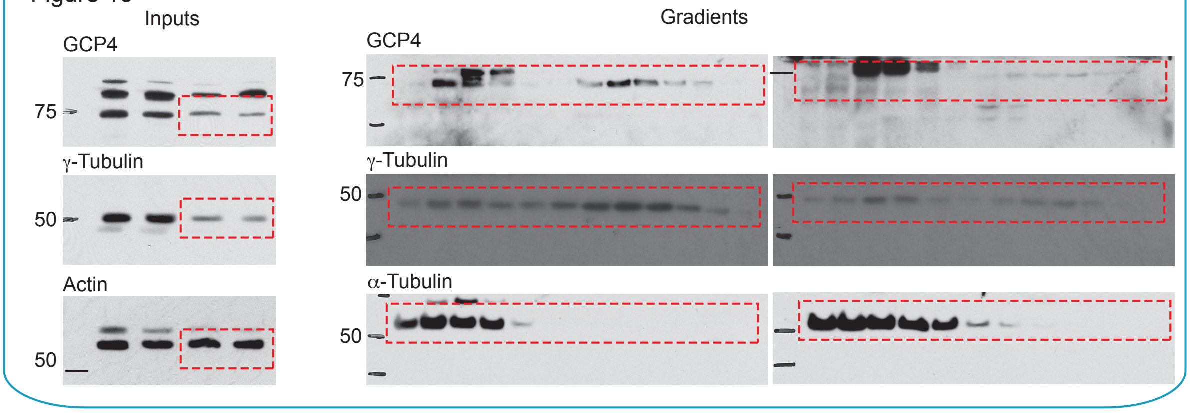

Figure 1d

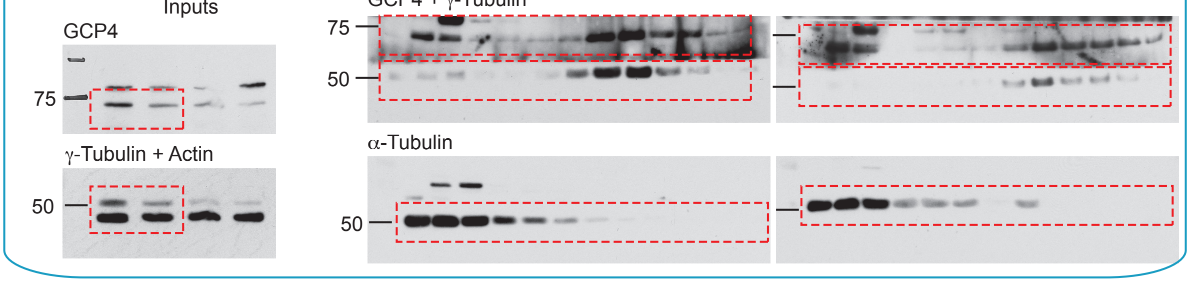

Figure 4a

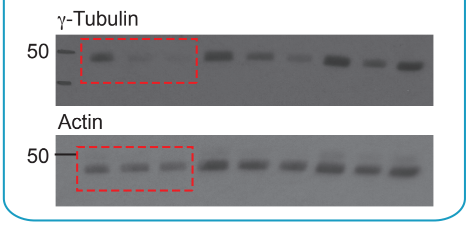

Figure 5a

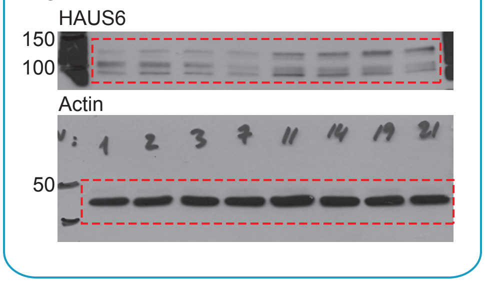

Figure 5c

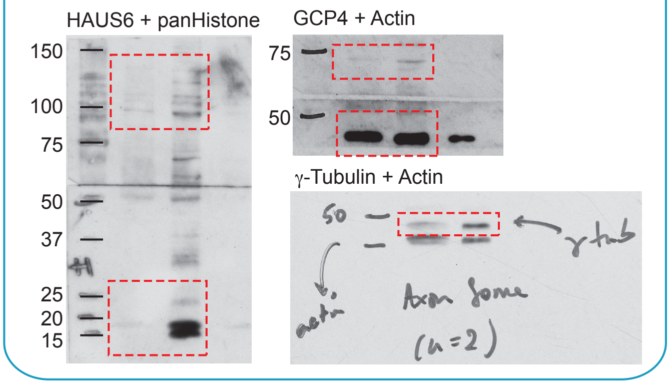

Figure 5b

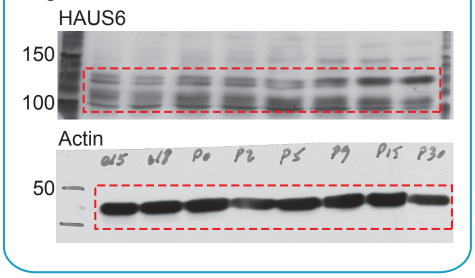

Figure 5d

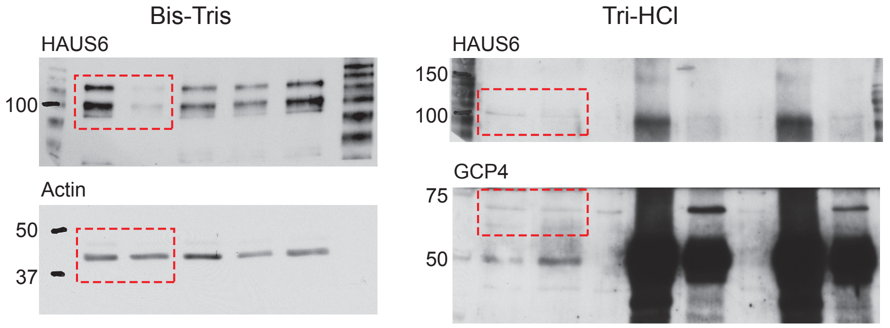

Figure 5e

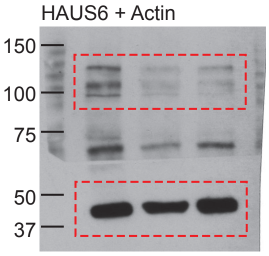

Figure 5f

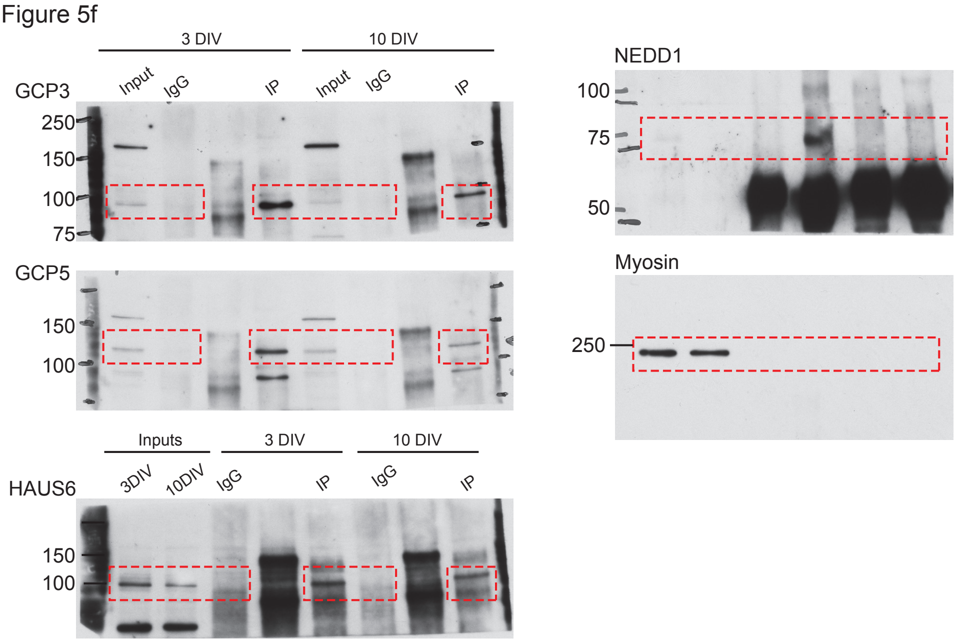

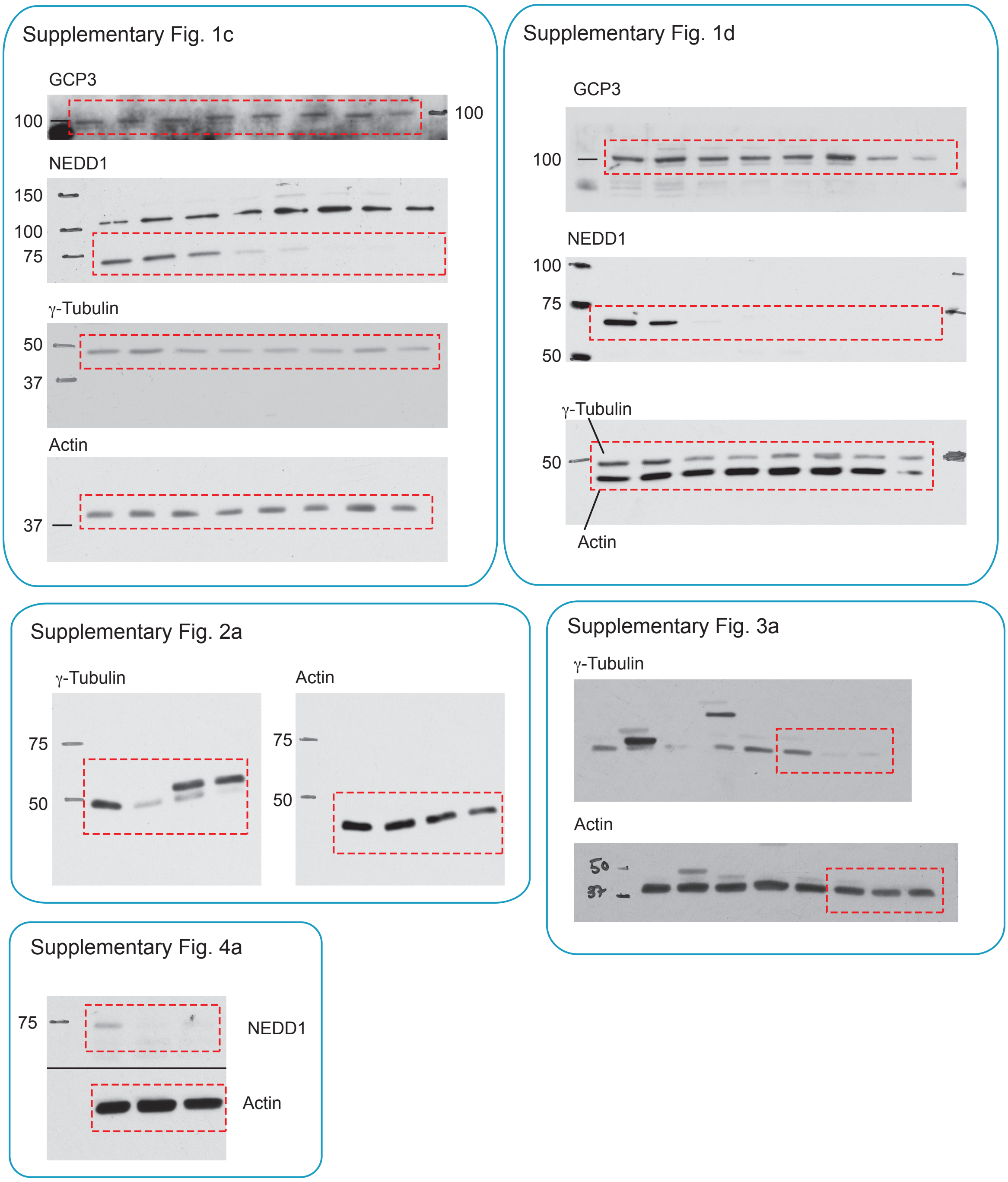

Supplementary Figure 7 Uncropped Western blots corresponding to Figures 1c,d, 4a, 5a,b,c,d,e,f, Supplementary Figures 1c, d, 2a, 3a and 4a .
